# B3GNT6-Linked Multimodal Signatures Integrate Tissue Morphology and PTM-Related Transcriptomics to Stratify Tumor

**DOI:** 10.7150/ijbs.134004

**Published:** 2026-05-18

**Authors:** Kun Mei, Yipeng Xu, Renjun Gu, Zilu Chen, Foxing Tan, Zhuyan Shao, Jiahong Zhu, Xu Wang, Xigao Liu, Yan Huang, Xiaoxuan Yu, Hengrui Liu

**Affiliations:** 1 Nanjing University of Chinese Medicine, Nanjing, China.; 2 Department of Oncology, The People's Hospital of Bozhou, the Affiliated Bozhou Hospital of Anhui Medical University, Bozhou, China.; 3 Department of Oncology, Zhejiang Cancer Hospital,Hangzhou, Zhejiang, China.; 4 School of Chinese Medicine, Nanjing University of Chinese Medicine, Nanjing 210023, China.; 5 Department of Gastroenterology and Hepatology, Jinling Hospital, Medical School of Nanjing University, Nanjing, 210002, China; 6 Department of Gynecology, The People's Hospital of Bozhou, the Affiliated Bozhou Hospital of Anhui Medical University, Bozhou, China.; 7 Department of Gynecologic Oncology, Zhejiang Cancer Hospital,Hangzhou, Zhejiang, China.; 8 Department of Urology, Qilu Hospital, Cheeloo College of Medicine, Shan dong university, Shandong, China.; 9 Department of Ultrasound, Nanjing Hospital of Chinese Medicine Affiliated to Nanjing University of Chinese Medicine, Nanjing, China.; 10 School of Medicine, Nanjing University of Chinese Medicine, Nanjing, China.; 11 State Key Laboratory of Pharmaceutical Biotechnology, School of Life Sciences, Nanjing University, Nanjing, China.; 12 Department of Biochemistry, University of Cambridge, UK.

**Keywords:** colon adenocarcinoma, multimodal integration, pathomics pathology, post-translational modifications, B3GNT6

## Abstract

Colon adenocarcinoma (COAD) exhibits substantial molecular and microenvironmental heterogeneity, limiting the reliability and clinical application of single-omics biomarkers. Post-translational modifications (PTMs) play a pivotal role in connecting genotype to phenotype, yet prognostic models informed by PTMs seldom incorporate tissue architecture alongside transcriptomic states. This study developed a PTM-based multimodal framework for prognostic stratification and mechanistic analysis in COAD. Additionally, bulk RNA-seq data, clinicopathological information, and whole-slide H&E images from TCGA-COAD were integrated with single-cell RNA-seq (GSE132465) and spatial transcriptomics (GSE225857). Differentially expressed genes related to PTMs were identified, and pathomic features derived from H&E slides were fused with transcriptomic data via an autoencoder-based latent space. Prognosis-related latent features were then selected to establish a patient-level PTM-related multimodal risk score (PTMLS). A total of 102 PTM-related differentially expressed genes were enriched in glycan biosynthesis and ubiquitin-mediated pathways. The PTMLS effectively stratified patients into high- and low-risk groups, with significant differences in overall survival in both training (HR = 3.54, 95% CI 2.67-4.70, P = 0.001) and validation cohorts (HR = 2.68, 95% CI 1.49-4.82, P = 0.006). Low-risk tumors displayed higher immune and ESTIMATE scores, lower TIDE scores, distinct immunotherapy responder profiles, and differential predicted drug sensitivity. Cross-modal interpretation identified B3GNT6 as a key contributor linked to histopathological features. Single-cell and spatial analyses localized B3GNT6-associated epithelial states and their interactions with vascular and stromal compartments. Functional experiments further revealed that B3GNT6 expression was downregulated in tumors and inhibited colorectal cancer proliferation, migration, tumor growth, and metastasis. This PTM-informed multimodal framework integrates routine pathology and transcriptomic data, providing robust prognostic stratification, clinically relevant immune and therapeutic phenotyping, and mechanistic insight supporting B3GNT6 as a potential therapeutic target in COAD.

## Introduction

Colorectal cancer, particularly colon adenocarcinoma, demonstrates significant heterogeneity in molecular subtypes, immune microenvironmental composition, and therapeutic responses, which directly impact clinical outcomes and the effectiveness of individualized treatments^1^, ^2^. While transcriptomic profiles, mutational landscapes, and immune-infiltration-based biomarkers are widely employed for risk stratification and response prediction, single-omics approaches often fail to capture the complete biological state of the tumor^3^. Intracellular signaling and immune evasion are critically dependent on dynamic regulation at the protein level, while the spatial architecture and microenvironmental context encoded in tissue morphology are often not fully recoverable from molecular measurements alone^4^.

Post-translational modifications (PTMs) regulate protein stability, subcellular localization, intermolecular interactions, and enzymatic activity, serving as a key effector layer that connects genotype to phenotype in tumorigenesis and immunoregulation^5^, ^6^. Disruptions in PTM-associated gene networks can reprogram malignant cell states and remodel the immune microenvironment by altering antigen presentation, cytokine signaling, metabolic pathways, and stress responses, ultimately influencing sensitivity to immunotherapy^7^, ^8^. However, most PTM-focused studies remain limited to individual genes or pathways, with few unified modeling frameworks that integrate molecular states, tissue morphology, and microenvironmental responses across scales. Similarly, robust risk-stratification tools that utilize routine clinical data—such as H&E histology and RNA sequencing—are scarce.

Recent advances in digital pathology and deep learning have enabled the extraction of quantitative histopathological features from H&E slides^9^, ^10^. Concurrently, single-cell RNA sequencing (scRNA-seq) and spatial transcriptomics provide unparalleled resolution for dissecting cellular composition, cell states, and their spatial organization. Despite these advances, differences in scale across modalities, discordant noise structures, and the curse of dimensionality still limit their effective use in survival modeling and clinical translation^11^. Thus, there is an urgent need for a comprehensive strategy that extracts synergistic representations from both histomorphological and transcriptomic data, minimizing reliance on a priori feature selection, and directly supporting prognostic and therapeutic-response predictions.

This study developed a COAD-specific multimodal integrative framework that combines deep pathomics features derived from histopathology with PTM-related transcriptomic signals to construct a joint feature matrix. An autoencoder was employed to denoise the data and learn low-dimensional latent representations. In this latent space, prognostically distinct subtypes were identified through survival-associated feature selection followed by unsupervised clustering. Subsequently, an individualized risk score was derived to construct a PTM-related multimodal risk system (PTMLS) for patient stratification. The associations between PTMLS-defined risk groups and the immune microenvironment, immunotherapy response surrogates (e.g., TIDE), tumor mutational burden (TMB), immune-phenotype atlases, and predicted drug sensitivity were systematically evaluated. By integrating single-cell and spatial transcriptomic data, this study localized key molecular drivers and formulated mechanistic hypotheses. Notably, features like B3GNT6 were identified as highly influential contributors in both transcriptomic and deep histopathological domains, suggesting that molecular programs and tissue phenotypes jointly define a prognostic risk axis. Through spatial expression mapping, compartment-level organization, and pseudo-knockdown perturbation analyses of single-cell gene regulatory networks (SCGRNs), this study proposed a B3GNT6-associated epithelial state and its interactions with the microenvironment as a plausible regulatory framework. This provides a biological rationale and translational context for PTM-informed risk stratification.

## Materials and Methods

### Data collection and processing

scRNA-seq data were obtained from the GEO database (GSE132465), while bulk RNA-seq data from TCGA were consolidated into a COAD-specific dataset. Only COAD patients with complete clinical information, high-quality pathology slide images, and corresponding RNA-seq expression data from TCGA were included in the analysis. Samples with missing metadata, poor image resolution, or ambiguous histological classification were excluded. Spatial transcriptomics (stRNA-seq) data were sourced from GSE225857, and pathology learning images were obtained from the TCGA database. Additionally, clinicopathological information and whole-genome expression data from immunotherapy cohorts (GSE126044 and GSE78220) were also incorporated. For a comprehensive overview of the dataset characteristics and PTMRG documentation, please refer to the supplementary tables S1.

### Identification and evaluation of a PTMLS

The limma R package was used for PTMRG expression profiling between tumor and adjacent normal tissues, with differentially expressed genes (DEGs) identified using the criteria of FDR < 0.05 and |log_2_ fold change (FC)| > 1. Differential expression analysis was performed with the edgeR package to identify genes that play a key role in PTMs.

### Multi-scale feature analysis based on ResNet-50 and CellProfiler

To leverage the diagnostic potential and real-world accessibility of routine histopathology for COAD, a multi-scale feature-learning workflow was developed to capture both tissue-level morphology and cell-resolved phenotypes. H&E whole-slide scans for TCGA-COAD cases were obtained from The Cancer Genome Atlas and generated at 20 × scanning resolution. After standardized quality control, each slide was partitioned into 512 × 512 pixel patches without overlap. Non-informative regions were excluded using an Otsu-derived tissue mask, retaining only patches with a tissue area fraction exceeding 0.80. To mitigate inter-slide staining heterogeneity, all selected patches underwent Macenko color normalization prior to downstream processing.

For deep representation learning, normalized patches were processed using OpenCV, converted from BGR to RGB, and standardized with ImageNet-style intensity normalization (mean = [0.485, 0.456, 0.406]; SD = [0.229, 0.224, 0.225]). Patches were resized to 224 × 224 pixels and processed through a pretrained ResNet-50 model implemented in PyTorch. Activations from the final convolutional stage (layer4) were extracted, resulting in a 2048 × 7 × 7 tensor per patch. Spatial max pooling across the 7 × 7 grid reduced this tensor into a 2048-element vector that retained high-level histomorphological features—such as glandular organization, stromal patterning, and nuclear architecture—for subsequent machine learning modeling and survival analysis.

Simultaneously, quantitative cellular descriptors were obtained using CellProfiler (v4.0.7). RGB images were stain-deconvolved with the UnmixColors module using optical-density separation to generate hematoxylin- and eosin-enriched channels. Nuclei were segmented on the hematoxylin channel via the IdentifyPrimaryObjects module with adaptive thresholding and diameter constraints (10-50 pixels), and whole-cell regions were delineated by outward propagation using the IdentifySecondaryObjects module. Intensity metrics (MeasureObjectIntensity), texture statistics (MeasureTexture), morphometric attributes (MeasureObjectSizeShape), and intensity-distribution profiles (MeasureObjectIntensityDistribution) were computed. After curating the features to remove redundant or low-utility measurements, 11 image-derived variables were retained for downstream analyses. All AI-enabled steps, including feature extraction and model development, adhered to the TITAN Guidelines 2025 to ensure transparency and reproducibility.

### Integrated multimodal framework

An end-to-end multimodal framework was developed, integrating histology-derived pathomics and transcriptomic measurements into a unified sample-by-feature table. Prior to model input, histopathological images were standardized and transformed into numerical representations compatible with neural network learning. Model training utilized a multilayer feedforward neural network with three hidden layers consisting of 128, 64, and 32 neurons, respectively; a dropout rate of 0.1 was applied to mitigate overfitting. Optimization was achieved through batch gradient descent, with a batch size of 64, a learning rate of 0.001, and 150 training epochs. To facilitate cross-platform representation learning without extensive feature pruning, an autoencoder was trained, where the encoder projected the high-dimensional multi-omics input into a compact latent space, and the decoder reconstructed the original profiles to impose denoising and stability constraints. This approach mitigated dimensionality challenges and enhanced integrative modeling by encouraging biologically meaningful embeddings suited for downstream survival prediction and subtype identification.

Using the caret package, the TCGA cohort was randomly split into training and validation sets at a 7:3 ratio. Each latent coordinate (or its corresponding reconstructed feature contribution) was assessed for prognostic relevance via univariate Cox proportional hazards regression. Latent factors that met a predefined significance threshold were retained for subtype discovery. Gaussian mixture modeling was then applied to cluster patients within the selected latent space, with the optimal number of clusters determined by maximizing the silhouette coefficient to balance separation, cohesion, and model simplicity. The identified groups exhibited distinct overall-survival trajectories, indicating that the learned multimodal representation captured clinically relevant heterogeneity. A patient-specific risk index was then computed, and the cohort was stratified into high- and low-risk categories using the median risk as the cutoff, with Kaplan-Meier curves quantifying survival differences between strata.

### Genomic alterations and immune landscape analyses

TMB was calculated using the maftools R package. To estimate potential sensitivity to immune checkpoint blockade, TIDE scores and immunotherapy response likelihood were predicted across PTMLS-defined subgroups using the TIDE algorithm. Immunogenomic annotations from The Cancer Immunome Atlas (TCIA) were retrieved to further characterize immune phenotypes and identify potential responders. Immune infiltration and pathway activity were assessed via single-sample gene set enrichment analysis (ssGSEA), focusing on lymphocyte-related signatures and immune programs. Concurrently, intratumoral immune cell composition was inferred using multiple deconvolution methods from the IOBR framework, providing robust, cross-validated estimates of immune cell abundances.

### Computational pipeline for single-cell transcriptome analysis

Raw transcriptomic count matrices were processed using Seurat (v4.3.2) for quality control and normalization. Genes expressed in fewer than 10 cells within each sample were discarded. Cells were retained if they met standard QC criteria, excluding low-complexity or potentially damaged profiles (fewer than 200 or more than 5,000 detected genes) and removing cells with excessive mitochondrial content (>10% of total UMIs). Batch effects across samples were harmonized using the Harmony algorithm to correct for technical variation. Dimensionality reduction was performed by PCA on highly variable genes, and expression structure was visualized with UMAP using the top 30 principal components. Marker genes for each cell population were identified through differential expression testing with Seurat's FindAllMarkers function.

### Spatial transcriptomics analysis

For spatial transcriptomics, low-quality spots with very low total UMI counts or disproportionately high mitochondrial transcripts were excluded. Data preprocessing was performed in Seurat, utilizing SCTransform (SCT) for normalization followed by unsupervised clustering to delineate spatial domains. Cell-type identities were assigned by integrating H&E morphology with cluster-enriched marker genes. Spatial gene expression landscapes were visualized using Seurat's SpatialDimPlot and SpatialFeaturePlot, providing an integrated view of tissue architecture and regional transcriptional programs.

### Pseudotime analysis

Differentiation continua were reconstructed from scRNA-seq data using Monocle2. After dimensionality reduction and pseudotime ordering under standard settings, cells were arranged along putative developmental paths, allowing for the tracking of progressive state changes within a low-dimensional manifold. To further corroborate and refine these inferences, CytoTRACE was applied. This tool independently estimates developmental potential based on the established decline in transcriptional complexity during maturation. CytoTRACE was run with default parameters, providing an orthogonal differentiation score that complemented the Monocle2 pseudotime results and enhanced the robustness of the trajectory findings.

### Single-cell virtual knockout

To model an in silico knockout of B3GNT6 in single cells, this study utilized scTenifoldKnk (R, v1.0.2) and applied it to wild-type (WT)/control CRC scRNA-seq profiles to derive gene-specific perturbation signatures. Epithelial/tumor cells were processed using the package's default quality filters (including mitochondrial read fraction and minimum library size) and normalized by CPM-based scaling within the standard workflow.

A stable WT scGRN was constructed by repeatedly subsampling cells and inferring multiple directed networks via principal component regression, retaining the most prominent gene-gene dependencies in each iteration. The resulting adjacency matrices were assembled into a tensor, which was then denoised and aggregated using CANDECOMP/PARAFAC (CP) decomposition to yield a robust consensus WT scGRN. For the pseudo-KO network, B3GNT6's regulatory influence was removed by setting all edges originating from B3GNT6 to zero weight in the WT adjacency structure. WT and pseudo-KO scGRNs were compared through nonlinear manifold alignment, embedding genes into a shared low-dimensional space. Perturbed targets were prioritized based on the Euclidean displacement between their WT and pseudo-KO embeddings. Statistical significance was assessed using Benjamini-Hochberg FDR control, defining significant perturbations at FDR < 0.05 unless otherwise noted. The significant perturbed gene set was further analyzed using pathway and gene-set enrichment analyses to infer the putative biological consequences of B3GNT6 loss in the CRC cellular context.

### CRC specimen acquisition

Between January 2025 and December 2025, paired CRC specimens and matched adjacent non-tumor tissues were obtained from five patients who underwent surgical resection at Nanjing Hospital of Chinese Medicine, Affiliated with Nanjing University of Chinese Medicine. All patients had pathologically confirmed CRC. The study was conducted in accordance with the Declaration of Helsinki and approved by the Ethics Committee of Nanjing Hospital of Chinese Medicine, Affiliated with Nanjing University of Chinese Medicine.

### Materials and cell transfection

All reagents and antibodies used are listed in Supplementary Table S2. The human CRC cell lines HT-29, HCT116, SW480, SW620, and RKO were purchased from Procell (Wuhan, China) with STR authentication and confirmed to be mycoplasma-free prior to use.

For B3GNT6 gain- and loss-of-function studies, HCT116 cells were transduced with Puro-CMV-B3GNT6 (overexpression) or Puro-LV-shB3GNT6 (knockdown) vectors (Shanghai Genechem, China). Viral supernatants were collected 72 hours after transfection, concentrated using a precipitation-based method according to the System Biosciences protocol, and delivered into CRC cells using TUNDUX transduction reagents. Stable transductants were selected via puromycin resistance. The same procedure was applied to HT-29 cells.

### *In vitro* functional experiments

Cell proliferation was assessed using colony formation and CCK-8 assays, while cell motility was evaluated by Transwell and wound-healing experiments. Flow cytometry was performed to analyze cell-cycle distribution and apoptosis. All procedures followed established protocols as previously reported^15^.

### Western blot

Protein concentrations from RIPA-lysed cells and tissues were quantified using a BCA assay. Equal amounts of protein were separated on 10% SDS-PAGE gels, then transferred to nitrocellulose membranes. Membranes were blocked with BSA (Servicebio, China) for 15 minutes, incubated with the specified primary antibodies overnight, and probed with appropriate secondary antibodies. Signals were visualized using an Amersham Imager 680 (Cytiva, USA).

### Immunofluorescence (IF) analysis

For IF, CRC tissue sections were incubated with primary antibodies at 4 °C overnight, followed by incubation with corresponding secondary antibodies for 2 hours at 37 °C in light-protected conditions. Nuclei were counterstained with DAPI for 10 minutes, and fluorescent signals were captured using a Nikon A1R confocal microscope (Japan).

### Animal experiments

Animal experiments were approved and supervised by the Animal Welfare and Ethics Committee of Nanjing University of Chinese Medicine. Eight-week-old male Balb/c nude mice, obtained from Cyagen, were randomly assigned to different groups, with each group containing at least six mice (n = 6). The xenograft model was established by subcutaneously injecting 1 × 10^6^ HT-29 and HCT-116 cells into BALB/c nude mice. Tumor dimensions were measured and recorded at seven-day intervals. After three weeks, the mice were euthanized, xenografts were excised and weighed, and immunohistochemistry (IHC) analysis was performed using specific antibodies. Liver metastasis models were generated by intravenously injecting 3 × 10^5^ luciferase-labeled HT-29 cells into the spleens of BALB/c nude mice. Metastasis was monitored using bioluminescence imaging at seven-day intervals with the NightOWL LB 983 system (Bertold, Germany). After the three-week period, the mice were euthanized, and livers were collected, photographed, and stained with hematoxylin and eosin (H&E) for metastatic lesion evaluation.

### Statistical analysis and data processing

Statistical analyses and figure generation were performed using R (v4.3.2). Associations between continuous variables were assessed using Spearman's rank correlation, while χ² tests were applied for categorical variables. Group differences were evaluated using either the Wilcoxon rank-sum test or two-tailed Student's t-test, depending on distributional assumptions. All analyses were conducted using default package settings, with a two-sided P value < 0.05 considered statistically significant.

## Results

### Variant landscape of post-translational modifications genes in COAD patients

**Figure 1:**
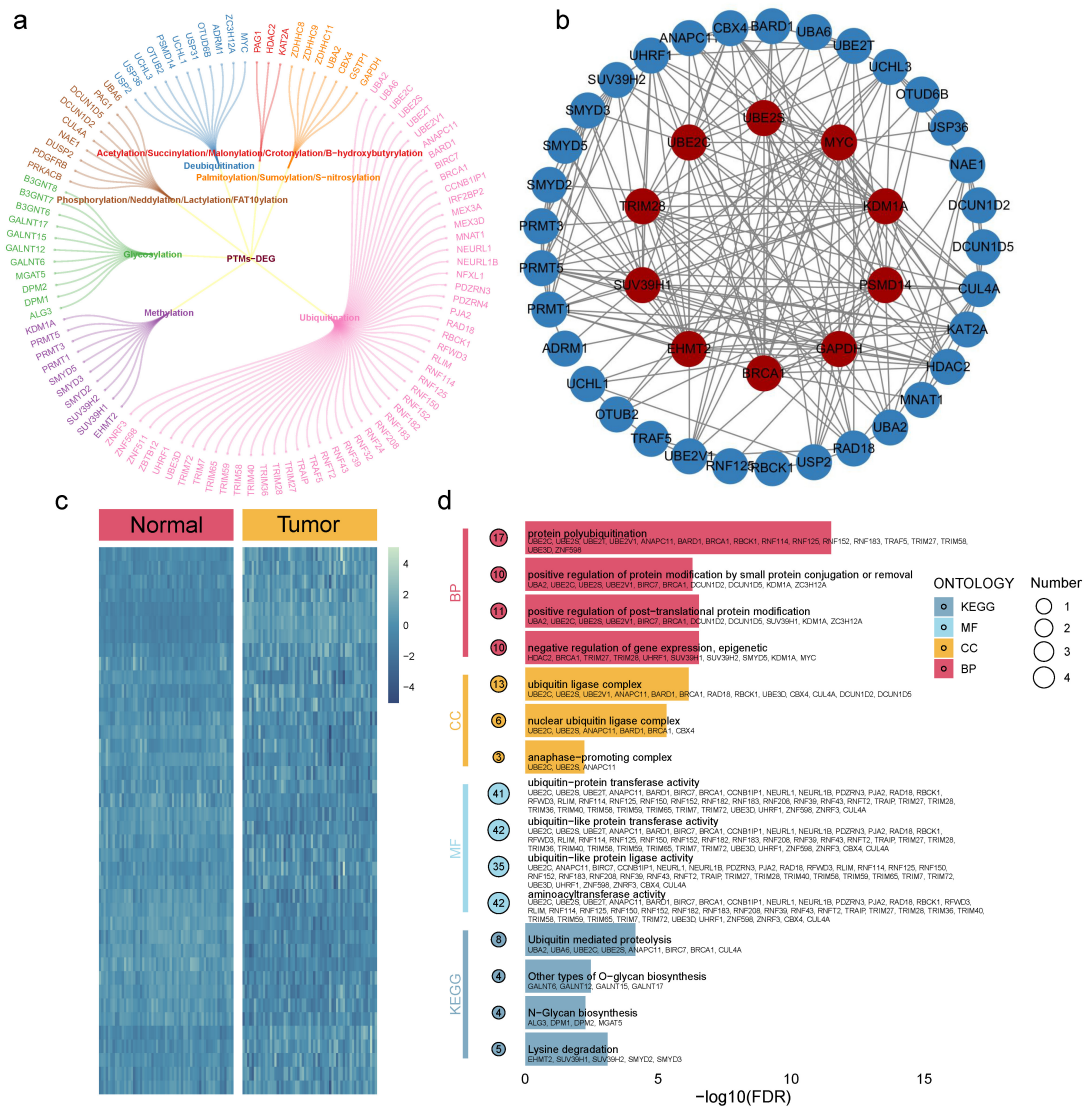
** Molecular landscape of PTMs.** a. The PTM-DEGs gene list includes 102 genes. b. PPI network depicting interactions among the PTM-DEGs. c. Heatmap comparing the expression of PTM-DEGs between COAD and normal tissues. d. GO and KEGG enrichment analyses based on the PTM-DEGs.

From the TCGA-COAD cohort, 102 DEGs were identified with statistical significance (all adjusted p < 0.05, log2FC > 1). These included 54 from ubiquitination, 11 from glycosylation, 10 from deubiquitination, 10 from methylation, 4 from neddylation, 3 from palmitoylation, 3 from phosphorylation, 2 (PAG1 and KAT2A) from succinylation, 2 (GSTP1 and GAPDH) from S-nitrosylation, 2 (UBA2 and CBX4) from sumoylation, and 1 (HDAC2) from acetylation (Fig. 1a). Protein-protein interaction (PPI) networks for these DEGs are shown in Fig. 1b, while heatmaps displaying the scaled RNA expression levels of DEGs are provided in Fig. 1c. Additionally, GO and KEGG enrichment analyses revealed that these DEGs are involved in multiple carcinogenesis-related pathways, including N-glycan biosynthesis, ubiquitin-mediated proteolysis, and O-glycan biosynthesis (Fig. 1d).

### A multimodal integration framework for identifying survival-associated subtypes and dissecting key driving features

**Figure 2:**
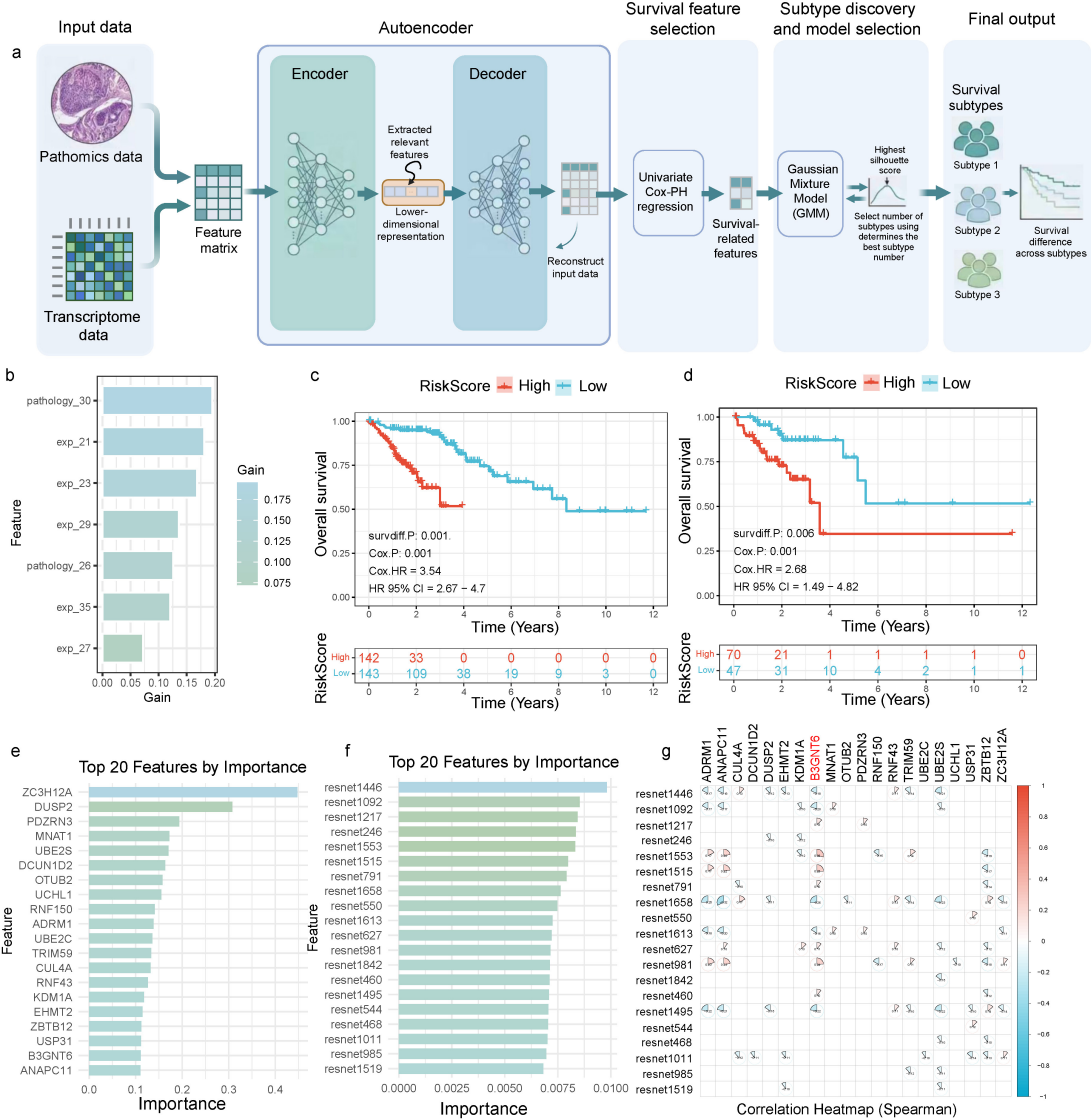
** Integrative pathomics-transcriptomics framework for survival stratification and prognostic feature identification.** a. Pathomics and transcriptomics features are integrated into a unified matrix, which is embedded using an autoencoder. Survival-associated dimensions/features are identified through univariate Cox proportional hazards regression, followed by Gaussian mixture model clustering; the optimal number of clusters is determined by maximizing the silhouette score, yielding distinct survival subtypes. b. Feature importance analysis reveals a concentrated contribution from the top-ranked features. c. Kaplan-Meier analysis of the training cohort, stratified by risk score, shows significant separation between high- and low-risk groups (P = 0.001), with a hazard ratio (HR) of 3.54 (95% CI 2.67-4.70). d. Validation cohort reproduces the prognostic stratification (P = 0.006), with HR = 2.68 (95% CI 1.49-4.82). e-f. Top 20 transcriptomic and deep pathomics (ResNet-derived) features ranked by importance. g. Spearman correlation heatmap of selected key features highlights correlated modules and potential redundancy/coordination.

A multimodal prognostic subtyping framework was developed that integrates pathomics features derived from pathology images and transcriptomic data into a unified feature matrix. An autoencoder was employed to generate a low-dimensional latent representation, thereby reducing redundancy and emphasizing cross-modal concordant signals (Fig. 2a). Within the latent space, univariable Cox proportional hazards regression was applied to identify dimensions/features significantly associated with survival, followed by unsupervised subtyping on these prognosis-enriched representations. Patient subtypes were determined using Gaussian mixture model (GMM) clustering, with the optimal number of clusters selected by maximizing the silhouette coefficient, resulting in subtypes with distinct survival outcomes. To interpret the drivers of risk stratification, feature contributions were assessed using gain/importance metrics, revealing a pronounced head-concentrated pattern that indicated prognostic information is predominantly provided by a small subset of highly informative features (Fig. 2b), such as pathology_30 and exp_21.

The resulting risk score demonstrated stable prognostic stratification in both the training and validation cohorts (Fig. 2c-2d). In the training set (Fig. 2c), overall survival significantly differed between high- and low-risk groups (two-sided log-rank test, P = 0.001), and Cox regression revealed a substantially higher event risk in the high-risk group (two-sided Cox model, HR = 3.54, 95% CI 2.67-4.70). In the validation set (Fig. 2d), this stratification was independently validated (two-sided log-rank test, P = 0.006; two-sided Cox model, HR = 2.68, 95% CI 1.49-4.82). The direction of effect was consistent, and confidence intervals did not cross 1, supporting the generalizability across cohorts rather than overfitting to the training data.

Notably, the top contributing features originated from two complementary evidence streams—transcriptomics and histomorphology. Both the top transcriptomic features and ResNet-derived deep histopathology phenotype features ranked among the most important, suggesting that molecular states and tissue morphology jointly shape the prognostic risk axis (Fig. 2e-2f). A Spearman correlation heatmap further revealed a modular correlation structure among key features (Fig. 2g). In particular, B3GNT6 showed strong correlations with multiple deep histopathology phenotypes (ResNet1553, Spearman = 0.28; ResNet1515, Spearman = 0.25), providing a structural explanation for the concentrated importance pattern and suggesting potential synergy and redundant encoding across features—thereby motivating downstream mechanistic mapping and redundancy-reduction validation.

### Risk-score stratification is associated with the immune microenvironment, therapeutic response, and drug sensitivity

**Figure 3:**
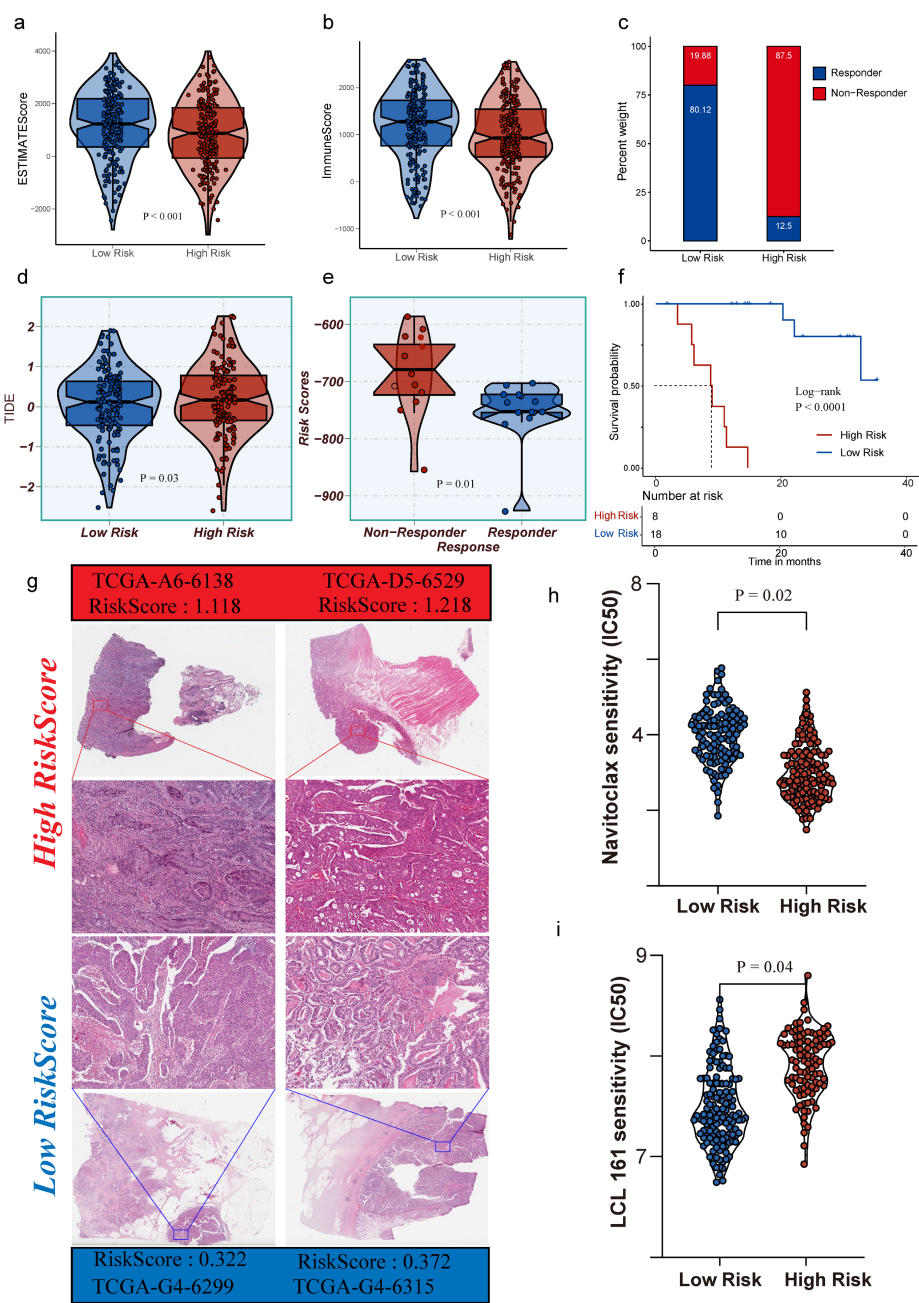
** Risk stratification links immune microenvironment, therapeutic response surrogates, survival outcome, and drug sensitivity.** a-b. Violin plots of ESTIMATE score and Immune score comparing low-risk and high-risk groups (two-sided, P < 0.001). c. Proportions of responders and non-responders across risk groups. d. TIDE scores stratified by risk group. e. Distribution of risk scores in responders versus non-responders (two-sided, significance as indicated). f. Kaplan-Meier overall survival curves for high-risk versus low-risk groups (two-sided log-rank test, P < 0.0001); numbers at risk are shown below. g. Representative histological images from TCGA samples in each risk group, with corresponding sample IDs and risk scores. h-i. Predicted drug sensitivity metrics differ between risk groups (two-sided, P = 0.024 and P = 0.047).

Risk stratification showed consistent associations with tumor microenvironmental states. Compared to the high-risk group, the low-risk group exhibited significantly higher ESTIMATE and Immune scores (Fig. 3a-3b; two-sided, P < 0.001), indicating greater enrichment of immune and stromal components in the low-risk population. Similar directional differences were observed in immunotherapy response-related metrics (Fig. 3c-3e). Stratification by response status revealed that the proportions of Responders versus Non-responders supported a systematic link between risk grouping and treatment response (Fig. 3c). The distribution of risk scores differed significantly between Responders and Non-responders (Fig. 3e, P = 0.01), and TIDE scores were higher overall in the high-risk group (Fig. 3d, P = 0.03), which correlated with a phenotype of increased immune evasion and lower likelihood of benefiting from immunotherapy.

Survival analysis confirmed that the risk score provides robust and stable stratification of patient outcomes (Fig. 3f). Kaplan-Meier curves showed significantly worse overall survival in the high-risk group compared to the low-risk group (two-sided log-rank test, P < 0.0001), with clear separation maintained throughout the follow-up period. Histological analysis of TCGA H&E slides revealed increased tumor lymphocyte infiltration in the low-risk group (Fig. 3g). These results support PTMLS as a promising biomarker for predicting immune response and guiding therapeutic stratification. In addition to immunity and survival, risk stratification also corresponded to differences in treatment sensitivity (Fig. 3h-3i). Two drug-sensitivity indices showed significant differences between the high- and low-risk groups (two-sided, P = 0.02 and P = 0.04), suggesting potential utility in treatment selection or the design of combination strategies. In clinical practice, this raises the possibility that PTMLS-based stratification could help prioritize patients for immunotherapy-based regimens or support the selection of combined or non-immunotherapy treatments for patients predicted to have limited responsiveness to immune checkpoint blockade. Collectively, these results position PTMLS as a promising biomarker for immunotherapy response prediction and treatment stratification.

### B3GNT6 is downregulated in colon adenocarcinoma and is associated with adverse survival, immune features, and therapy-related transcriptional programs

**Figure 4:**
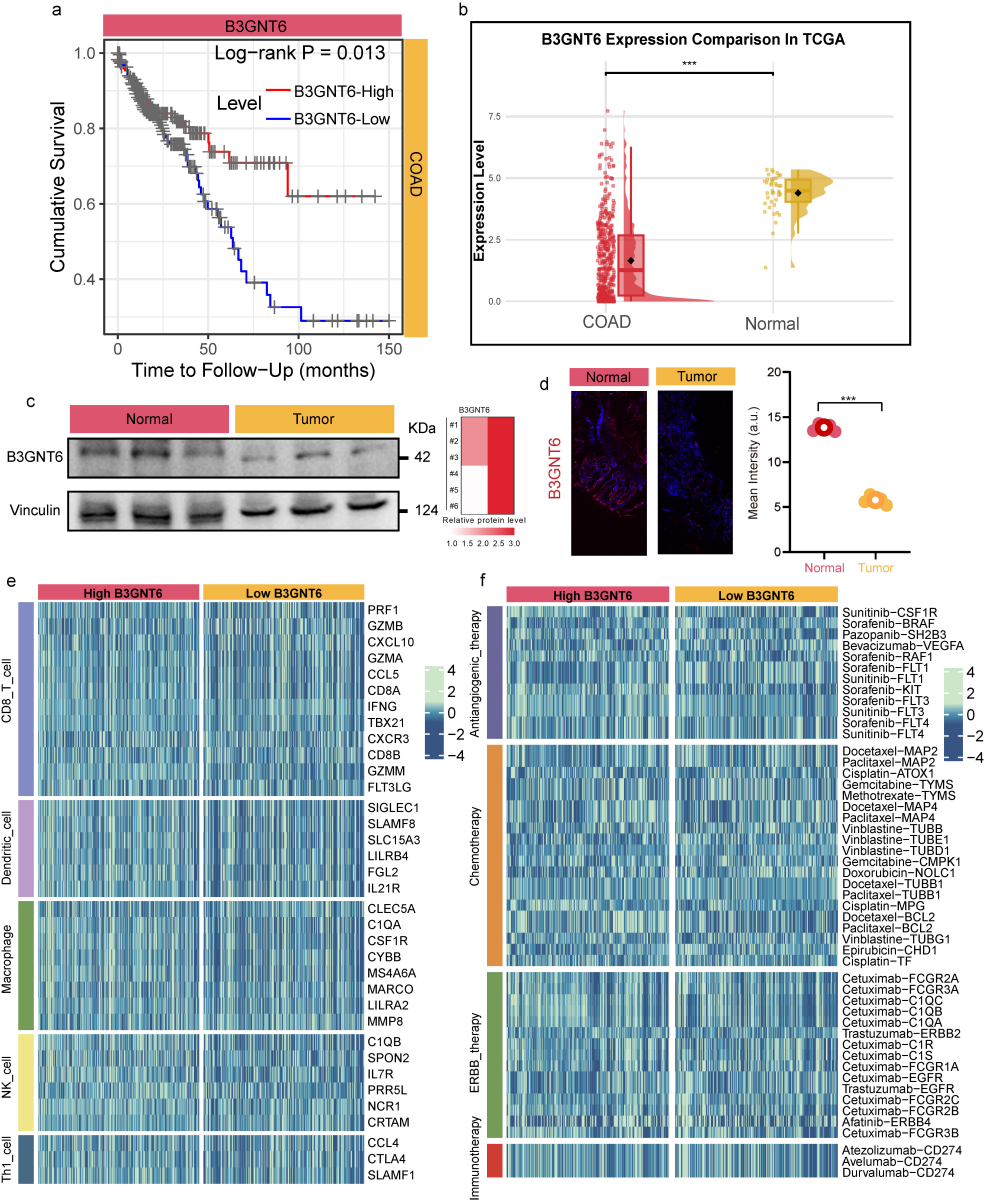
** B3GNT6 upregulation in COAD correlates with poor survival, immune signatures, and therapy-related profiles.** a. Kaplan-Meier overall survival stratified by B3GNT6 expression (two-sided log-rank test, P = 0.013). b. B3GNT6 mRNA expression in TCGA-COAD tumors versus normal tissues (two-sided; significance indicated by *). c. Western blot of B3GNT6 in clinical COAD specimens (tumor vs normal), with Vinculin as loading control and quantification shown. d. Immunofluorescence of B3GNT6 in clinical COAD specimens (tumor vs normal) with corresponding intensity quantification. e. Heatmaps of immune cell-related gene expression signatures comparing high versus low B3GNT6 groups across indicated immune cell types. f. Heatmaps of therapy-associated gene/signature profiles comparing high versus low B3GNT6 groups across indicated therapeutic categories.

The clinical relevance of B3GNT6 in COAD and its potential associations with immunity and therapeutic response were assessed. Kaplan-Meier analysis revealed a significant difference in overall survival between expression-defined groups, with patients exhibiting high B3GNT6 expression having poorer prognoses (two-sided log-rank test, P = 0.013; Fig. 4a), suggesting that B3GNT6 is linked to unfavorable clinical outcomes. At the transcript level, TCGA data indicated that B3GNT6 is significantly downregulated in COAD tumor tissues (Fig. 4b). This finding was independently corroborated at the protein and tissue levels in clinical COAD samples. Western blotting demonstrated reduced B3GNT6 band intensity in tumor tissues compared to paired normal controls, with consistent quantitative differences across replicate samples (Fig. 4c). IF staining further confirmed enhanced B3GNT6 expression in normal tissues (Fig. 4d).

Stratified analyses further implicated B3GNT6 in tumor immune states and therapeutic responsiveness. A heatmap of immune cell-related genes showed distinct immune gene expression profiles between high- and low-B3GNT6 groups across several immune cell types (e.g., CD8 T cells, dendritic cells, macrophages, NK cells, Th1 cells) (Fig. 4e), supporting a systematic association with immune infiltration and functional states. Similarly, a therapy-related signal heatmap revealed stratified differences between high- and low-B3GNT6 groups across multiple treatment axes—anti-angiogenic therapy, chemotherapy, ERBB-targeted therapy, and immunotherapy—based on corresponding gene/marker patterns (Fig. 4f). These results suggest that B3GNT6 is not only prognostically significant but may also be involved in the biological contexts underlying therapeutic sensitivity and immune response to immunotherapy. Additionally, this study assessed the generalizability of B3GNT6 across various cancers and observed a consistent benign trend in both solid and non-solid tumors (Supplementary Figure S1).

### Single-cell mapping localizes the cellular source of B3GNT6 and reveals epithelial-state transcriptional programs and potential trajectories

**Figure 5:**
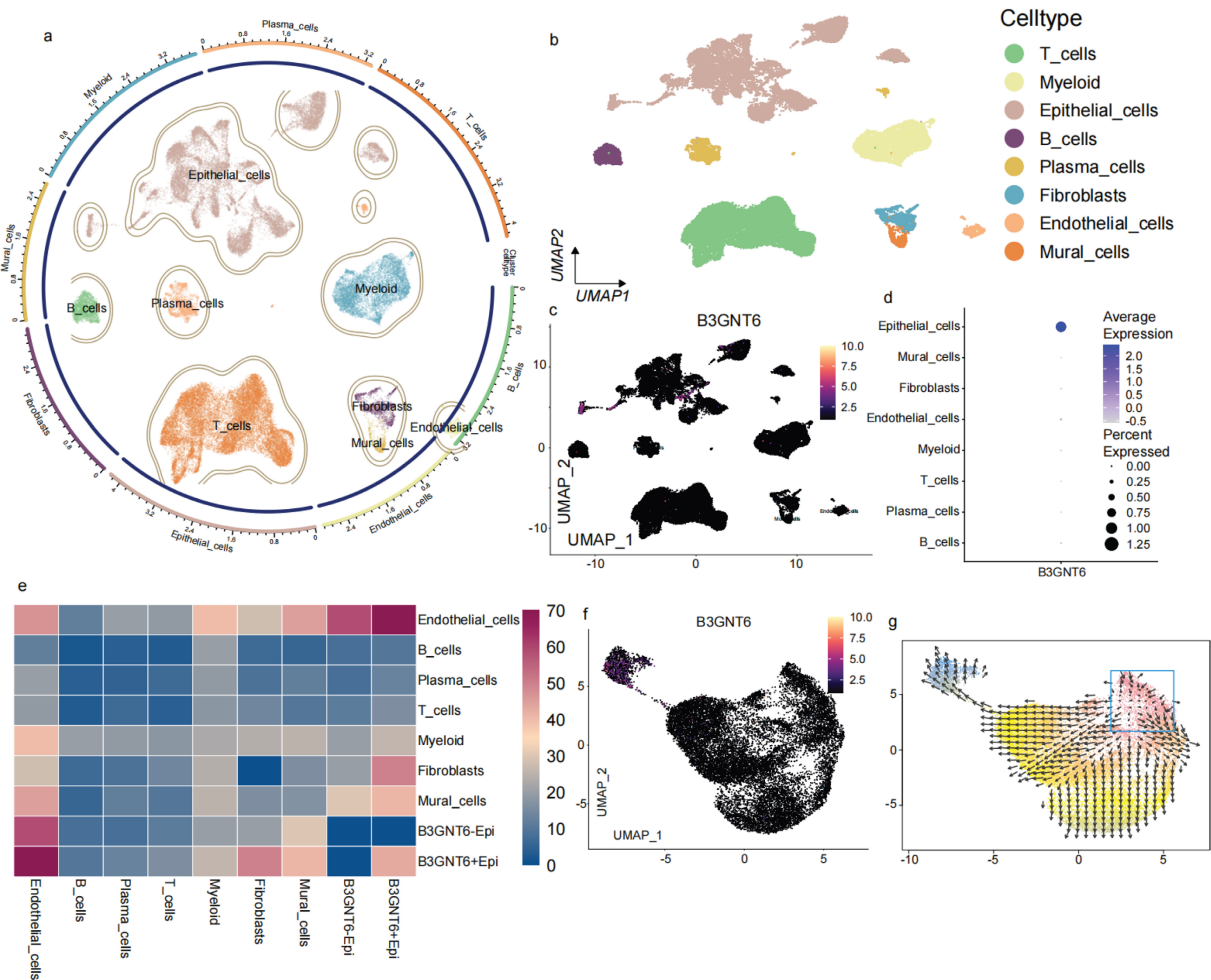
** Single-cell atlas identifies B3GNT6-expressing cellular compartments and delineates B3GNT6-associated epithelial states.** a. Circular UMAP embedding of all single cells with cluster boundaries and annotated major cell compartments. b. UMAP visualization of the same embedding colored by annotated cell types (T cells, Myeloid cells, Epithelial cells, B cells, Plasma cells, Fibroblasts, Endothelial cells, Mural cells). c. Feature plot showing B3GNT6 expression projected onto the UMAP embedding (color intensity indicates relative expression). d. Dot plot summarizing B3GNT6 expression across annotated cell types; dot color indicates average expression, and dot size represents the percentage of cells expressing B3GNT6 (percent expressed). e. Heatmap of relative gene expression across indicated cell types/epithelial states, highlighting transcriptional programs associated with B3GNT6-related epithelial subpopulations (e.g., B3GNT6-Epi and B3GNT6+Epi). f. UMAP feature plot highlighting B3GNT6-associated epithelial subclusters/states within the epithelial compartment (B3GNT6-Epi vs B3GNT6+Epi). g. RNA velocity field overlaid on the UMAP embedding, indicating directional transitions among cellular states and providing dynamic context for B3GNT6-associated epithelial trajectories.

Single-cell transcriptomic profiling with cell-type annotation revealed clear separation of major cell populations, identifying eight distinct subsets: T cells, Myeloid cells, Epithelial cells, B cells, Plasma cells, Fibroblasts, Endothelial cells, and Mural cells (Fig. 5a-5b). Gene-level visualization of expression on UMAP showed a pronounced bias in the distribution of B3GNT6 across cell types, with epithelial cells representing the primary expressing subset (Fig. 5c-5d). At a finer resolution, a heatmap further divided epithelial cells into B3GNT6-associated states (e.g., B3GNT6-Epi and B3GNT6+Epi), revealing state-specific expression patterns and a modular structure across cell groups (Fig. 5e). Notably, both B3GNT6-Epi and B3GNT6+Epi states were strongly associated with endothelial cells, suggesting a potential role of B3GNT6 in epithelial-endothelial transitions.

Finally, vector field/trajectory direction plots suggested directional state transitions (Fig. 5f-5g). Arrows in UMAP space inferred the directions of state migration, indicating that certain regions may represent earlier or more initiating states extending toward other cell groups or states. These observations support the interpretation of B3GNT6-associated epithelial programs within a dynamic cellular-state landscape, rather than as a purely static differential expression pattern.

### Spatial transcriptomics reveals the tissue distribution of B3GNT6-associated epithelial states and their spatial coupling to vascular/stromal compartments

**Figure 6:**
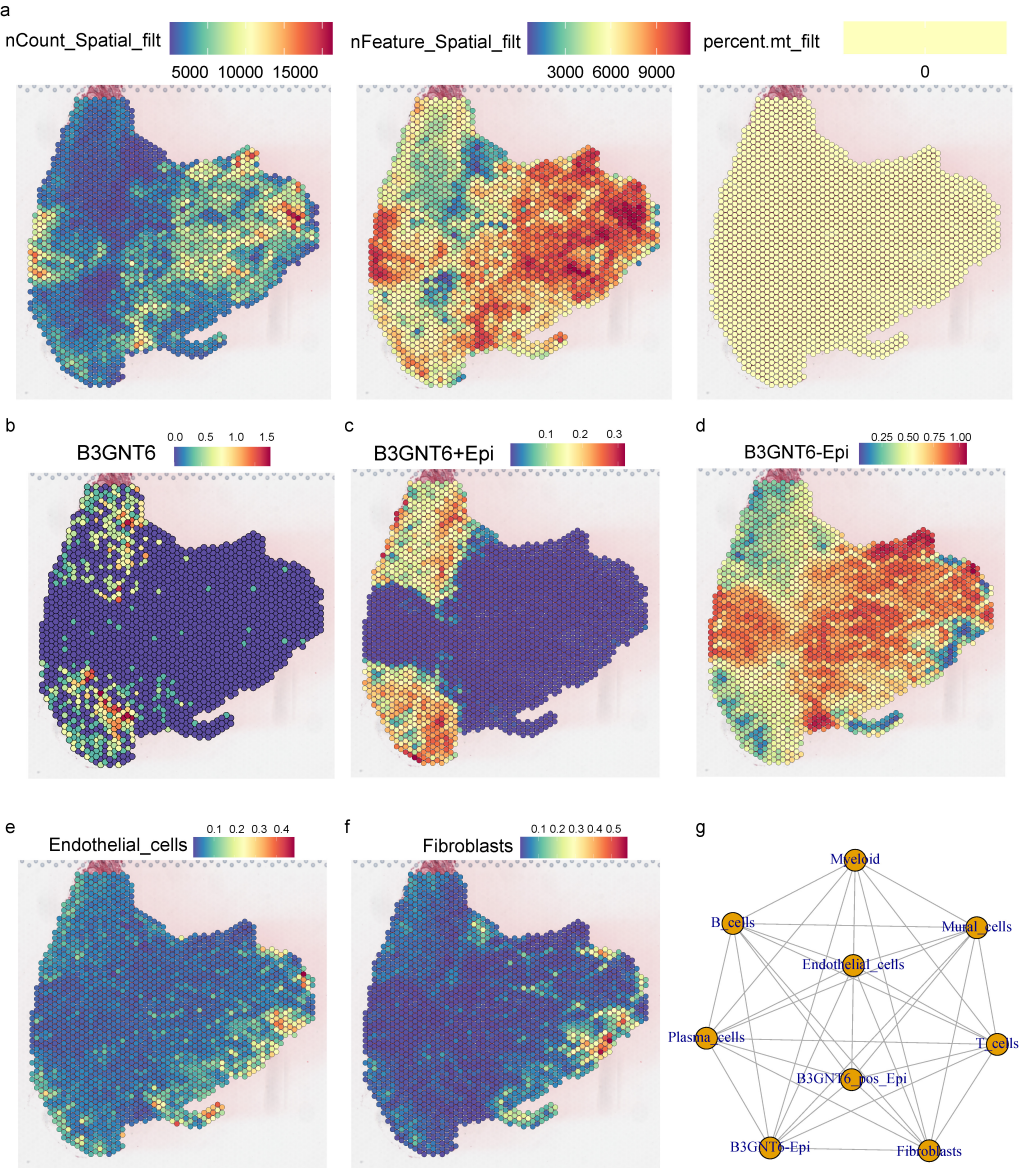
** Spatial transcriptomics maps B3GNT6-associated epithelial states and their spatial coupling with stromal and vascular compartments.** a. Spatial quality-control maps showing nCount_Spatial_filt, nFeature_Spatial_filt, and percent.mt_filt across spots. b. Spatial feature plot of B3GNT6 expression. c. Spatial score/expression map for B3GNT6+Epi (B3GNT6-associated epithelial state). d. Spatial score/expression map for B3GNT6-Epi (complementary epithelial state). e. Spatial distribution/score of Endothelial cells. f. Spatial distribution/score of Fibroblasts. g. Inferred cell-cell interaction network among indicated compartments, including B3GNT6-associated epithelial states and microenvironmental cell types.

Spatial transcriptomics quality-control metrics demonstrated consistent sequencing depth and gene detection across tissue regions: nCount_Spatial_filt and nFeature_Spatial_filt remained high within valid tissue areas, while mitochondrial proportion (percent.mt_filt) was relatively uniform without abnormal elevations (Fig. 6a), supporting the reliability of downstream spatial expression mapping and cell-composition analysis. Spatially, B3GNT6 displayed marked regional enrichment, rather than a uniform distribution (Fig. 6b). Further decomposition of epithelial states revealed distinguishable spatial patterns for B3GNT6+Epi and B3GNT6-Epi within the tissue (Fig. 6c-6d), suggesting that B3GNT6 reflects not merely an expression-level shift but an epithelial transcriptional state with defined anatomical localization. Cell-type spatial scores further elucidated microenvironmental compartmentalization: Endothelial cells and Fibroblasts formed characteristic enriched regions (Fig. 6e-6f), showing spatial proximity and interface relationships with B3GNT6-associated epithelial states. This indicates that these epithelial states may arise within specific vascular and stromal contexts, providing histological coordinates for interpreting the coupling between epithelial states and the microenvironment.

Cell-cell interaction networks also revealed potential communication links across multiple cell types (Fig. 6g). Notably, B3GNT6-associated epithelial populations exhibited visible interaction edges with stromal/vascular and immune-related cell groups, reinforcing its role as a key node in microenvironmental signaling exchange.

### Pseudo-knockdown analysis of single-cell regulatory networks reveals downstream regulatory axes and pathway enrichment for B3GNT6

**Figure 7:**
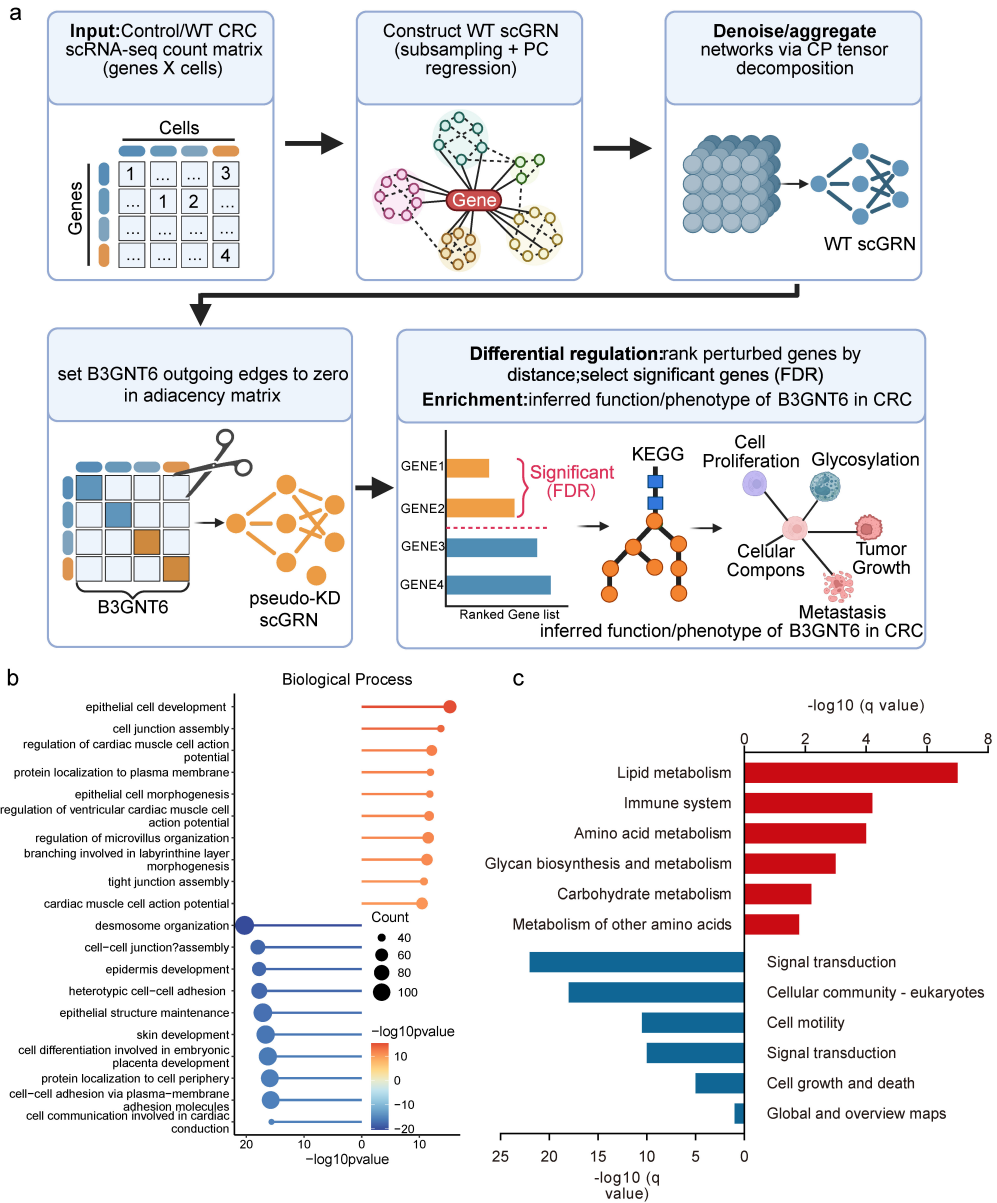
** Pseudo-knockdown perturbation of scGRN delineates B3GNT6 downstream programs in CRC.** a. Workflow for constructing WT scGRN from the WT CRC scRNA-seq count matrix (subsampling and PC regression), denoising/aggregation via CP tensor decomposition, and generating a pseudo-KD scGRN by setting all outgoing edges of B3GNT6 to zero. Differentially regulated targets are prioritized based on network proximity to B3GNT6 and statistical significance (two-sided testing with Benjamini-Hochberg FDR correction; threshold as defined in the analysis). b. Biological process of pseudo-KD-responsive genes, visualized as -log10(q-value) (FDR-adjusted; two-sided enrichment test). Red/blue bars indicate processes positively/negatively associated with the pseudo-KD perturbation, as indicated in the panel. c. Pathway enrichment of pseudo-KD-responsive genes, visualized as -log10(q-value) (FDR-adjusted; two-sided enrichment test). Red/blue bars indicate pathways positively/negatively associated with the pseudo-KD perturbation, as indicated in the panel.

An scGRN was constructed using WT CRC single-cell transcriptomic data. Initial regulatory adjacency was derived through subsampling and principal component regression, followed by denoising and network aggregation using CP (CANDECOMP/PARAFAC) tensor decomposition to enhance robustness (Fig. 7a). A pseudo-knockdown (pseudo-KD) perturbation was simulated by setting all outgoing edges of B3GNT6 to zero, resulting in a pseudo-KD scGRN. Candidate downstream genes were ranked and selected based on network distance and differential regulatory signals.

Differentially regulated genes under pseudo-KD showed coherent expression and regulatory responses, with both upregulated and downregulated genes identified in log_2_FC, indicating that B3GNT6 influences multiple parallel regulatory branches. Pathway enrichment analyses revealed that both positively and negatively enriched pathways converge on B3GNT6 as an upstream node, with the potential to systematically remodel cell junction assembly, epithelial cell development, glycan biosynthesis and metabolism, as well as cell growth and death, contributing to its functional role in CRC (Fig. 7b-7c).

### B3GNT6 suppresses CRC cell proliferation and migration *in vitro*

**Figure 8:**
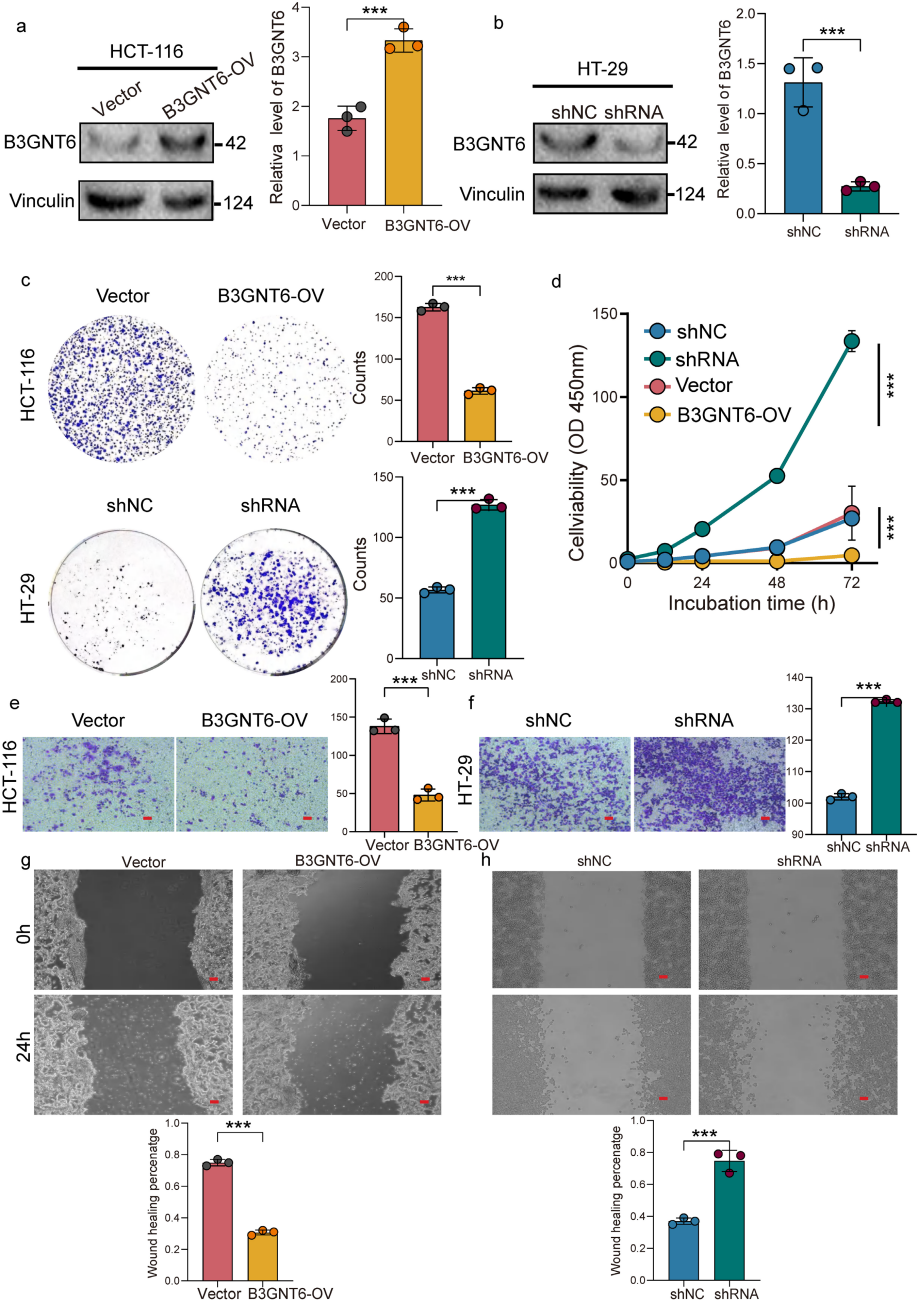
** B3GNT6 restrains colorectal cancer cell proliferation and migration in gain- and loss-of-function assays.** a. Western blot confirming B3GNT6 overexpression (B3GNT6-OV) in HCT-116 cells versus vector control; Vinculin as loading control with quantification. b. Western blot confirming B3GNT6 knockdown in HT-29 cells (shRNA) versus shNC control; Vinculin as loading control with quantification. c. Colony formation assays showing reduced clonogenic growth upon B3GNT6 overexpression in HCT-116 and increased clonogenic growth upon B3GNT6 knockdown in HT-29; representative images and quantification. d. Cell viability/proliferation curves over indicated incubation times (0-72 h) demonstrating decreased growth with B3GNT6-OV in HCT-116 and increased growth with B3GNT6 knockdown in HT-29. e. Transwell migration assays indicating suppressed migration with B3GNT6-OV in HCT-116 and enhanced migration with B3GNT6 knockdown in HT-29; representative fields and quantification. f. Wound-healing assays showing reduced wound closure with B3GNT6-OV in HCT-116 and accelerated closure with B3GNT6 knockdown in HT-29; quantification shown. Statistical significance is denoted as indicated in the panels.

The mRNA and protein expression of B3GNT6 were assessed in multiple CRC cell lines. Our results showed that B3GNT6 expression was relatively elevated in HT-29 cells, while it was significantly reduced in HCT-116 cells (Supplementary Figure S2). HCT-116 cells were subsequently modified to stably overexpress B3GNT6 for gain-of-function analysis (Fig. 8a). In contrast, two shB3GNT6 constructs were transfected into HT-29 cells to suppress B3GNT6 expression. ShRNA1, demonstrating superior knockdown efficiency, was selected for subsequent loss-of-function studies (Fig. 8b, Supplementary Figure S3). After confirming B3GNT6 expression by western blotting, the effects on cell viability and proliferation were evaluated using CCK8 and colony formation assays. B3GNT6 overexpression inhibited the proliferation of HCT-116 cells, while B3GNT6 knockdown increased proliferative activity in HT-29 cells (Fig. 8c-8d). Furthermore, cell migration assays, including Transwell and wound healing experiments, revealed that B3GNT6 overexpression suppressed the migratory capabilities of HCT-116 cells, while B3GNT6 downregulation enhanced the migratory abilities of HT-29 cells (Fig. 8e-8f).

### B3GNT6 suppresses CRC cell proliferation and migration *in vivo*

**Figure 9:**
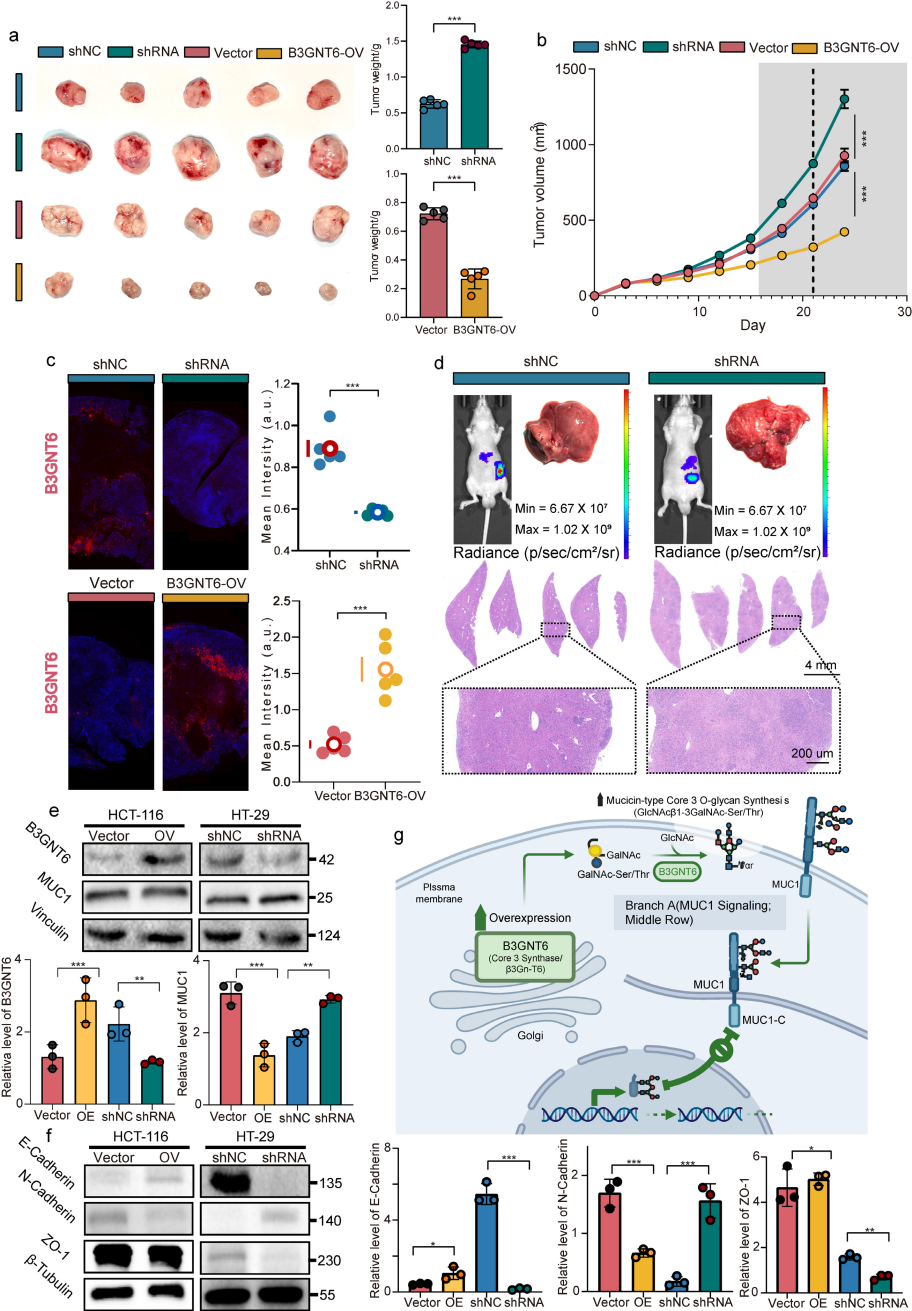
** B3GNT6 suppresses CRC tumor growth *in vivo* and is linked to MUC1-associated mucin-type Core 3 O-glycosylation.** a. Representative images of excised xenograft tumors from indicated groups (shNC vs shRNA; Vector vs B3GNT6-OV) and corresponding tumor weight quantification. b. Tumor weight change over time during tumor growth in each group. c. Fluorescent staining of B3GNT6 in different groups. d. *In vivo* bioluminescence imaging showing longitudinal tumor burden changes across groups. *Ex vivo* analyses including tumor bioluminescence and representative histology of tumor tissues. e. Western blot analysis of B3GNT6 and MUC1 protein expression in HCT116 cells with B3GNT6 overexpression and HT29 cells with B3GNT6 knockdown. Vinculin was used as a loading control. f. Western blot analysis of EMT-related proteins, including E-cadherin, N-cadherin, and ZO-1, in HCT116 cells with B3GNT6 overexpression and HT29 cells with B3GNT6 knockdown. β-Tubulin was used as a loading control. Quantification of relative protein levels is shown on the right. Statistical significance is denoted as indicated in the panels. g. Schematic illustration of the proposed mechanism by which B3GNT6 suppresses colorectal cancer progression. B3GNT6 may regulate mucin-type O-glycosylation of MUC1, reduce MUC1 protein expression, and inhibit EMT-related molecular changes, as evidenced by increased E-cadherin and ZO-1 and decreased N-cadherin, thereby attenuating tumor growth and metastasis. Quantification of relative protein levels is shown on the right. Data are presented as mean ± SD from three independent experiments. *P < 0.05, **P < 0.01, ***P < 0.001.

To investigate B3GNT6's impact on tumor growth and metastasis *in vivo*, shB3GNT6, B3GNT6-OV, and control cells were injected into BALB/c nude mice to establish xenograft and liver metastasis models. Xenograft model results revealed that B3GNT6 overexpression significantly reduced tumor growth rates and tumor weights in the B3GNT6-OV group compared to controls at the study endpoint. In contrast, B3GNT6 knockdown in the shB3GNT6 group markedly increased the tumor growth rate, resulting in a substantial increase in tumor weight (Fig. 9a-9b). Similarly, liver metastasis models demonstrated that metastatic inhibition was significantly diminished in the shB3GNT6 group. H&E staining of liver sections further validated these findings (Fig. 9c-9d).

Given the inhibitory effects of B3GNT6 on tumor growth and metastasis in vivo, we next explored whether B3GNT6 regulates MUC1 expression and EMT-related protein changes in colorectal cancer cells. Western blot analysis demonstrated that overexpression of B3GNT6 in HCT116 cells markedly decreased MUC1 protein expression. In parallel, B3GNT6 overexpression significantly upregulated E-cadherin and ZO-1, while downregulating N-cadherin. In contrast, B3GNT6 knockdown in HT29 cells led to the opposite expression pattern, characterized by increased MUC1 and N-cadherin levels together with reduced E-cadherin and ZO-1 expression (Fig. 9e-f). Collectively, these results suggest that B3GNT6 exerts a tumor-suppressive role by repressing MUC1 expression and inhibiting EMT-like molecular alterations, thereby contributing to the attenuation of colorectal cancer progression and metastasis.

## Discussion

The PTMLS framework presented here integrates pathomorphology and transcriptome-derived PTM-related signals into a unified latent space, combining two traditionally isolated sources of evidence. This approach facilitates robust prognostic stratification and biologically interpretable subtype identification in COAD. Compared to single-omics risk models, the strength of multimodal representation learning is twofold. First, under reconstruction constraints, the autoencoder generates low-dimensional embeddings that are stable, denoised, and internally consistent, thereby minimizing the overfitting risk associated with high-dimensional features. Second, feature-importance rankings highlight both top transcriptomic features and ResNet-derived deep histopathological phenotypes as key contributors, revealing a modular correlation structure. This suggests that tumor molecular programs and histomorphological changes are not independent, but rather converge along a shared risk axis. Such cross-modal concordance bolsters model transferability and clinical applicability. While a formal benchmark against conventional clinical-variable-based and single-omics models was beyond the scope of this study, the PTMLS's ability to jointly model histomorphological and PTM-related transcriptomic information presents a significant advantage over traditional methods that capture only one aspect of tumor biology.

However, it is important to note that PTMs occur at the protein level, whereas the PTM-related transcriptomic signatures in this study serve as RNA-level proxies for PTM regulatory programs. These signatures reflect the transcriptional activity of enzymes, readers, erasers, substrates, and regulatory factors associated with PTMs, but they do not directly quantify protein modification abundance, site occupancy, or enzymatic activity. Therefore, while PTMLS provides a valuable framework for integrating PTM-related regulatory information with histopathological phenotypes, future studies incorporating proteomics, phosphoproteomics, glycoproteomics, or other PTM-specific datasets are necessary to directly validate the inferred PTM states.

The positive correlation between the PTMLS risk axis and immune infiltration suggests that this axis likely reflects a key ecological gradient in CRC: immune-accessible/immune-responsive versus immune-excluded/stroma-barrier states^12^, ^13^. This interpretation aligns with the CMS framework for CRC, where stroma-enriched subtypes are often associated with immune exclusion and poor outcomes, while immune-activated subtypes are characterized by immune entry and favorable prognosis. Consistently, this study observed higher Immune/ESTIMATE scores in the low-risk group, along with more prominent tumor-infiltrating lymphocytes (TILs) on H&E sections—results that are directionally consistent with multicenter evidence showing that the Immunoscore is a strong predictor of recurrence and survival in colon cancer. Conceptually, PTMLS can thus be viewed as a tool for compressing molecular and morphological information into a shared immune-ecological axis, providing a common framework for clinical signals that are otherwise difficult to reconcile (e.g., morphological infiltration, transcriptomic immunity, and prognosis)^14^-^16^.

In contrast to immune entry, immune escape and exclusion can be computationally delineated. The elevated TIDE scores observed in the high-risk group provide a mechanistically informative interpretation of the risk axis from an immune perspective. TIDE quantifies immune evasion through two primary mechanisms: induction of T-cell dysfunction in the presence of high cytotoxic T lymphocyte infiltration, or inhibition of T-cell entry in the context of low cytotoxic T lymphocyte infiltration^17^. This aligns with the well-documented phenomenon of stromal immune exclusion in the tumor microenvironment. For instance, TGFβ-driven stromal programs can restrict T-cell infiltration into the tumor core and impair the efficacy of PD-(L)1 blockade; in CRC models, TGFβ-associated microenvironments have been identified as key determinants of immune-cold phenotypes and immune escape^18^. Consequently, a more comprehensive interpretation of the current findings suggests that PTMLS distinguishes distinct immune-evasive ecological niches, rather than directly predicting immunotherapy benefit in COAD. Validation in CRC-specific immune checkpoint blockade cohorts is necessary before clinical utility in immunotherapy selection can be established.

The identified PTM-related differentially expressed genes (PTM-DEGs) were enriched in pathways such as N-/O-glycan biosynthesis and ubiquitin-mediated proteolysis, consistent with an oncogenic landscape in which proteostasis remodeling and glycan reprogramming jointly influence tumor invasion and immune regulation. In the context of glycosylation, systematic reviews have emphasized its multifaceted roles in tumor progression, including cell-cell and cell-matrix interactions, receptor signaling, and immunomodulation, while also highlighting its translational potential as a source of clinically actionable biomarkers and therapeutic targets. Therefore, the value of PTMLS extends beyond statistical performance; it connects immune entry failure and tissue architectural remodeling to more proximal, potentially targetable effector networks.

The observed directional tension for B3GNT6 in COAD—downregulation in tumors relative to normal tissues, tumor-suppressive effects in *in vivo*/*in vitro* assays, and variable prognostic associations in tumor stratifications—likely reflects the composite nature of bulk expression data. Population-average tumor-normal differences can coexist with significant intratumoral heterogeneity. Notably, B3GNT6 encodes Core 3 β1,3-N-acetylglucosaminyltransferase, which plays a pivotal role in mucin-type O-glycan biosynthesis. Core 3 O-glycan synthesis is intimately associated with mucosal barrier homeostasis^19^. Mice lacking Core 3-derived O-glycans exhibit compromised mucus barriers and increased susceptibility to colitis and chemically induced colorectal tumorigenesis, suggesting a protective role for this axis in maintaining colonic epithelial integrity. In line with this, clinical studies have reported B3GNT6 downregulation in CRC, with lower expression linked to poorer survival outcomes, reinforcing a tumor-suppressive or protective role for B3GNT6^19^, ^20^.

B3GNT6, a key glycosyltransferase involved in the biosynthesis of Core 3 O-glycans, plays a critical role in epithelial homeostasis and mucin-type glycosylation. Increasing evidence suggests that its dysregulation contributes to cancer development and progression in a context-dependent manner. When higher B3GNT6 expression within specific tumor stratifications is associated with poorer outcomes, it is plausible that this elevated expression does not simply reflect intrinsic tumor-cell upregulation. Instead, it may signal the enrichment of particular cellular compositions, tissue architectures, or tumor-stroma interface niches. This phenomenon is common in bulk expression profiles. Studies like ESTIMATE have demonstrated that immune and stromal components can systematically perturb gene expression landscapes and correlate with tumor purity, suggesting that prognostic analyses should account for both purity and microenvironmental composition. Moreover, in colon cancer, stroma enrichment itself is a recognized adverse prognostic factor^21^. For example, the multicenter prospective UNITED study validated the tumor-stroma ratio as an independent prognostic factor, showing that a higher stromal proportion is associated with worse patient outcomes. Thus, interpreting B3GNT6 expression requires considering its context, integrating tumor-cell intrinsic effects with the broader tissue architecture and microenvironment.

Our functional experiments further highlight the tumor-suppressive role of B3GNT6 in CRC models. Overexpression of B3GNT6 inhibited CRC cell growth, migration, xenograft tumor growth, and metastatic colonization, while B3GNT6 knockdown promoted the opposite effects. Western blot analyses revealed that B3GNT6 perturbation altered the expression of MUC1 and key epithelial/cell-junction-associated markers, including E-cadherin, N-cadherin, and ZO-1. Specifically, B3GNT6 overexpression was associated with increased expression of epithelial and junctional markers, alongside reduced mesenchymal marker expression, while knockdown of B3GNT6 showed the opposite pattern. These results suggest that B3GNT6 may play a role in MUC1-associated regulation and in maintaining the epithelial state in CRC cells.

However, the mechanistic interpretation of the B3GNT6-MUC1 axis should be viewed as preliminary. Traditional Western blotting reveals total protein abundance but does not specifically measure distinct MUC1 glycoforms or Core 3 O-glycan structures. Therefore, while the present data support a potential link between B3GNT6 and MUC1 regulation, they do not establish a fully validated B3GNT6-Core 3 O-glycosylation-MUC1 pathway. Direct evidence from glycosylation-specific assays, such as lectin blotting, glycosylation-specific antibodies, mass spectrometry-based glycoproteomics, and rescue experiments, will be needed to validate this mechanism.

Several limitations should be acknowledged. First, the integration of heterogeneous platforms and data modalities—such as bulk transcriptomics, scRNA-seq, spatial transcriptomics, and digital pathology—may still introduce batch effects and treatment heterogeneity, potentially limiting generalizability despite efforts in representation learning and quality control. Second, variations in tumor types, treatment regimens, and sampling time points across immunotherapy cohorts may constrain direct inferences for COAD, necessitating larger, COAD-specific prospective real-world immunotherapy cohorts for validation. Third, clinical validation in this study was based on only five paired CRC specimens, which should be regarded as preliminary. While the findings align with computational and multi-omics results, the limited sample size may undermine the robustness of clinical confirmation. Fourth, although the autoencoder latent space improves fusion efficiency, its interpretability remains limited. Future efforts could incorporate more interpretable architectures—such as attention mechanisms, prototype learning, or causal representations—to enhance clinical communication. Finally, while spatial compartmentalization and interaction-network evidence highlight B3GNT6's biological importance, its direct role as a PTM-associated node, along with the relevant upstream and downstream pathways, requires clarification at the protein modification level.

In conclusion, PTMLS presents a scalable framework for multimodal integration. By leveraging routinely available H&E slides and transcriptomic data, it establishes a risk stratification system that is cohesively linked to prognosis, immune evasion, and potential therapeutic sensitivity. Moreover, the inclusion of single-cell and spatial data strengthens mechanistic inference by localizing key molecular states. This framework not only offers a novel tool for refined COAD subtyping and treatment stratification, but also provides a conceptual approach to understanding how PTM-related networks jointly shape tumor outcomes across multiple scales—from molecules to morphology to the microenvironment.

## Supplementary Material

Supplementary figures and tables.

## Data Availability

The data supporting the findings of this study are available from the corresponding author upon reasonable request. Furthermore, the datasets supporting the conclusions of this article are available in the NCBI Gene Expression Omnibus (GEO) repository: GSE132465 (https://www.ncbi.nlm.nih.gov/geo/query/acc.cgi?acc=GSE132465), GSE225857 (https://www.ncbi.nlm.nih.gov/geo/query/acc.cgi?acc=GSE225857), GSE126044 (https://www.ncbi.nlm.nih.gov/geo/query/acc.cgi?acc=GSE126044), and GSE78220 (https://www.ncbi.nlm.nih.gov/geo/query/acc.cgi?acc=GSE78220). Additionally, the dataset(s) supporting the conclusions of this article are available in the NCI Genomic Data Commons (GDC)/TCGA repository, TCGA-COAD (https://portal.gdc.cancer.gov/projects/TCGA-COAD). Additional datasets and materials that support the conclusions of this article are available upon reasonable request from the corresponding author.
