# Supplementary material for: B3GNT6-Linked Multimodal Signatures Integrate Tissue Morphology and PTM-Related Transcriptomics to Stratify Tumor
Source: Int J Biol Sci. 2026 May 18;22(10):5548–67. doi: 10.7150/ijbs.134004 (PMC13215456; doi:10.7150/ijbs.134004)
Supplement: Supplementary file 1 — Supplementary figures and tables. [file ijbsv22p5548s1.pdf]

**Supplementary Materials**

Supplementary Table S1. 20 types of post-translational modification gene sets.

| Acetylation | Succinylation | Ubiquitination |
|---|---|---|
| HAT1 | EP300 | UBA1 |
| AADAT | PAG1 | UBA2 |
| KYAT3 | KAT2A | UBA3 |
| GOT2 | CPT1A | UBA5 |
| KAT5 | SIRT5 | UBA6 |
| KAT6A | SIRT7 | UBA7 |
| KAT6B | Malonylation | AKTIP |
| KAT7 | EP300 | BIRC6 |
| KAT8 | PAG1 | UBE2A |
| ELP3 | KAT2A | UBE2B |
| GTF3C4 | SIRT5 | UBE2C |
| KAT14 | Crotonylation | UBE2D1 |
| EP300 | EP300 | UBE2D2 |
| PAG1 | PAG1 | UBE2D3 |
| EIF6 | KAT8 | UBE2D4 |
| KAT2B | KAT2A | UBE2E1 |
| SRC | SIRT1 | UBE2E2 |
| FGR | SIRT2 | UBE2E3 |
| NCOA3 | SIRT3 | UBE2E4P |
| ACAT1 | B-hydroxybutyrylation | UBE2F |
| BRD4 | EP300 | UBE2G1 |
| BRD3 | PAG1 | UBE2G2 |
| PBRM1 | SIRT3 | UBE2H |
| HDAC1 | HDAC1 | UBE2I |
| HDAC2 | HDAC2 | UBE2J1 |
| HDAC3 | Lactylation | UBE2J2 |
| HDAC4 | EP300 | UBE2K |
| HDAC5 | PAG1 | UBE2L1 |
| HDAC6 | HDAC1 | UBE2L2 |
| HDAC7 | HDAC3 | UBE2L3 |
| HDAC8 | Myristoylation | UBE2L4 |
| HDAC9 | NMT1 | UBE2L5 |
| HDAC10 | NMT2 | UBE2L6 |
| HDAC11 | SIRT6 | UBE2M |
| SIRT1 | ARF6 | UBE2N |
| SIRT2 | ARF1 | UBE2NL |
| SIRT3 | Sumoylation | UBE2O |
| SIRT4 | SAE1 | UBE2QL1 |
| SIRT5 | UBA2 | UBE2Q1 |
| SIRT6 | UBE2I | UBE2Q2 |
| SIRT7 | PIAS1 | CDC34 |

| | | |
|---|---|---|
| Palmitoylation | PIAS2 | UBE2R2 |
| ZDHHC1 | PIAS3 | UBE2S |
| ZDHHC2 | PIAS4 | UBE2T |
| ZDHHC3 | CBX4 | UBE2U |
| ZDHHC4 | RANBP2 | UBE2V1 |
| ZDHHC5 | ZNF451 | UBE2V2 |
| ZDHHC6 | SENP1 | UBE2W |
| ZDHHC7 | SENP2 | UBE2Z |
| ZDHHC8 | SENP3 | AFF4 |
| ZDHHC9 | SENP4 | AMFR |
| ZDHHC11 | SENP5 | ANAPC11 |
| ZDHHC12 | SENP6 | ANKIB1 |
| ZDHHC13 | SENP7 | AREL1 |
| ZDHHC14 | Neddylation | ARIH1 |
| ZDHHC15 | NAE1 | ARIH2 |
| ZDHHC16 | UBA3 | BARD1 |
| ZDHHC17 | UBE2M | BFAR |
| ZDHHC18 | UBE2F | BIRC2 |
| ZDHHC19 | RBX1 | BIRC3 |
| ZDHHC20 | RBX2 | BIRC7 |
| ZDHHC21 | CUL1 | BIRC8 |
| ZDHHC22 | CUL2 | BMI1 |
| ZDHHC23 | CUL3 | BRAP |
| LYPLA2P1 | CUL4A | BRCA1 |
| PPT1 | CUL4B | CBL |
| ABHD17A | CUL5 | CBLB |
| ABHD17B | CUL7 | CBLC |
| ABHD17C | DCUN1D1 | CBLL1 |
| ISGylation | DCUN1D2 | CCDC36 |
| UBE1L | DCUN1D3 | CCNB1IP1 |
| UBE2L6 | DCUN1D4 | CGRRF1 |
| HERC5 | DCUN1D5 | CHFR |
| HERC6 | NEDD8 | CNOT4 |
| TRIM25 | SENP8 | CUL9 |
| TRIM21 | CSN5 | CYHR1 |
| TRIM38 | CSN6 | DCST1 |
| USP18 | FAT10ylation | DTX1 |
| ATG8ylation | UBA6 | DTX2 |
| ATG7 | USE1 | DTX3 |
| ATG3 | RNF4 | DTX3L |
| ATG12 | HDAC6 | DTX4 |
| ATG5 | UFMylation | DZIP3 |
| ATG16L1 | UBA5 | E4F1 |
| MAP1LC3A | UFC1 | FANCL |

| | | |
|---|---|---|
| MAP1LC3B | UFL1 | G2E3 |
| MAP1LC3C | UFSP1 | HACE1 |
| GABARAP | UFSP2 | HECTD1 |
| GABARAPL1 | Glycosylation | HECTD2 |
| GABARAPL2 | ALG1 | HECTD3 |
| GABARAPL3 | ALG2 | HECTD4 |
| Methylation | ALG3 | HECW1 |
| SETD1A | ALG4 | HECW2 |
| SETD1B | ALG5 | HERC1 |
| EHMT1 | ALG6 | HERC2 |
| EHMT2 | ALG8 | HERC3 |
| SUV39H1 | ALG9 | HERC4 |
| SUV39H2 | ALG12 | HERC5 |
| SETDB1 | DPM1 | HERC6 |
| SETDB2 | DPM2 | HLTF |
| KMT2A | DPM3 | HUWE1 |
| KMT2B | DPAGT1 | IRF2BP1 |
| KMT2C | MGAT1 | IRF2BP2 |
| KMT2D | MGAT2 | IRF2BPL |
| SETD2 | MGAT3 | ITCH |
| PRDM9 | MGAT4 | KCMF1 |
| DOT1L | MGAT5 | KMT2C |
| NSD1 | OSTC | KMT2D |
| NSD2 | STT3A | LNX1 |
| NSD3 | STT3B | LNX2 |
| SETMAR | GALNT1 | LONRF1 |
| SMYD2 | GALNT2 | LONRF2 |
| SMYD3 | GALNT3 | LONRF3 |
| SMYD4 | GALNT4 | LRSAM1 |
| SMYD5 | GALNT5 | LTN1 |
| PRMT1 | GALNT6 | MAEA |
| PRMT2 | GALNT7 | MAP3K1 |
| PRMT3 | GALNT8 | 1-Mar |
| PRMT4 | GALNT9 | 10-Mar |
| PRMT5 | GALNT10 | 11-Mar |
| PRMT6 | GALNT11 | 2-Mar |
| PRMT7 | GALNT12 | 3-Mar |
| PRMT8 | GALNT13 | 4-Mar |
| PRMT9 | GALNT14 | 5-Mar |
| KDM1A | GALNT15 | 6-Mar |
| KDM1B | GALNT16 | 7-Mar |
| KDM2A | GALNT17 | 8-Mar |
| KDM2B | GALNT18 | 9-Mar |
| KDM3A | GALNT19 | MDM2 |

| | | |
|---|---|---|
| KDM3B | GALNT20 | MDM4 |
| KDM4A | GALNTL1 | MECOM |
| KDM4B | GALNTL2 | MEX3A |
| KDM4C | B3GNT1 | MEX3B |
| KDM4D | B3GNT2 | MEX3C |
| KDM5A | B3GNT3 | MEX3D |
| KDM5B | B3GNT4 | MGRN1 |
| KDM5C | B3GNT5 | MIB1 |
| KDM5D | B3GNT6 | MIB2 |
| KDM6A | B3GNT7 | MID1 |
| KDM6B | B3GNT8 | MID2 |
| KDM7A | B3GNT9 | MKRN1 |
| KDM7B | B4GALT1 | MKRN2 |
| Biotinylation | B4GALT2 | MKRN3 |
| BPL1 | B4GALT3 | MKRN4P |
| BPL2 | B4GALT4 | MNAT1 |
| S-nitrosylation | B4GALT5 | MSL2 |
| ADH5 | B4GALT6 | MUL1 |
| TXN | B4GALT7 | MYCBP2 |
| GSTP1 | | MYLIP |
| CASP3 | | NEDD4 |
| TXNRD1 | | NEDD4L |
| PDIA3 | | NEURL1 |
| GAPDH | | NEURL1B |
| Phosphorylation | | NEURL3 |
| AKT1 | | NFX1 |
| AKT2 | | NFXL1 |
| AKT3 | | NHLRC1 |
| MAPK1 | | NOSIP |
| MAPK3 | | NSMCE1 |
| PRKACA | | PARK2 |
| PRKACB | | PCGF1 |
| PRKACG | | PCGF2 |
| CAMK2A | | PCGF3 |
| CAMK2B | | PCGF5 |
| CAMK2D | | PCGF6 |
| CAMK2G | | PDZRN3 |
| SRC | | PDZRN4 |
| JAK1 | | PELI1 |
| JAK2 | | PELI2 |
| JAK3 | | PELI3 |
| TYK2 | | PEX10 |
| EGFR | | PEX12 |
| PDGFRB | | PEX2 |

| | |
|---|---|
| ABL1 | PHF7 |
| MEK1 | PHRF1 |
| MEK2 | PJA1 |
| PPP1CA | PJA2 |
| PPP1CB | PLAG1 |
| PPP1CC | PLAGL1 |
| PPP2CA | PML |
| PPP2CB | PPIL2 |
| PTPN1 | PRPF19 |
| PTPN11 | RAD18 |
| PTPRC | RAG1 |
| DUSP1 | RAPSN |
| DUSP2 | RBBP6 |
| DUSP3 | RBCK1 |
| Deubiquitination | RBX1 |
| USP7 | RC3H1 |
| USP20 | RC3H2 |
| CYLD | RCHY1 |
| USP15 | RFFL |
| USP13 | RFPL1 |
| USP4 | RFPL2 |
| OTUB1 | RFPL3 |
| USP17L2 | RFPL4A |
| USP33 | RFPL4AL1 |
| USP9X | RFPL4B |
| USP10 | RFWD2 |
| USP2 | RFWD3 |
| USP16 | RING1 |
| USP3 | RLF |
| USP37 | RLIM |
| USP19 | RMND5A |
| USP8 | RMND5B |
| USP12 | RNF10 |
| USP14 | RNF103 |
| USP1 | RNF11 |
| USP44 | RNF111 |
| VCPIP1 | RNF112 |
| USP11 | RNF113A |
| USP22 | RNF113B |
| USP28 | RNF114 |
| USP36 | RNF115 |
| BAP1 | RNF121 |
| WDR48 | RNF122 |
| USP21 | RNF123 |

| | |
|---|---|
| USP46 | RNF125 |
| OTUD7B | RNF126 |
| USP25 | RNF128 |
| BRCC3 | RNF13 |
| USP48 | RNF130 |
| USP5 | RNF133 |
| OTUD5 | RNF135 |
| USP24 | RNF138 |
| USP17L24 | RNF139 |
| USP17L25 | RNF14 |
| USP17L29 | RNF141 |
| USP17L30 | RNF144A |
| USP17L26 | RNF144B |
| USP17L27 | RNF145 |
| USP17L28 | RNF146 |
| USP47 | RNF148 |
| ABRAXAS2 | RNF149 |
| USP30 | RNF150 |
| UCHL3 | RNF151 |
| USP17L5 | RNF152 |
| USP26 | RNF157 |
| TNFAIP3 | RNF165 |
| YOD1 | RNF166 |
| USP49 | RNF167 |
| OTUB2 | RNF168 |
| UCHL5 | RNF169 |
| UBC | RNF17 |
| ATXN3 | RNF170 |
| USP17L6P | RNF175 |
| USP42 | RNF180 |
| USP17L1 | RNF181 |
| USP17L3 | RNF182 |
| USP35 | RNF183 |
| BABAM1 | RNF185 |
| EIF3F | RNF186 |
| USP51 | RNF187 |
| PSMD14 | RNF19A |
| ZRANB1 | RNF19B |
| UCHL1 | RNF2 |
| USP29 | RNF20 |
| USP34 | RNF207 |
| JOSD1 | RNF208 |
| BABAM2 | RNF212 |
| OTUD3 | RNF212B |

| | |
|---|---|
| USP27X | RNF213 |
| USP6 | RNF214 |
| USP38 | RNF215 |
| USP18 | RNF216 |
| USP17L22 | RNF217 |
| USP17L13 | RNF219 |
| USP17L18 | RNF220 |
| USP17L11 | RNF222 |
| USP17L17 | RNF223 |
| USP17L20 | RNF224 |
| USP17L10 | RNF225 |
| USP17L12 | RNF24 |
| USP17L19 | RNF25 |
| USP17L21 | RNF26 |
| ATXN7L3 | RNF31 |
| USP31 | RNF32 |
| JOSD2 | RNF34 |
| WDR20 | RNF38 |
| OTUD7A | RNF39 |
| OTUD6B | RNF4 |
| USP32 | RNF40 |
| OTUD1 | RNF41 |
| USP9Y | RNF43 |
| TP53 | RNF44 |
| STAMBPL1 | RNF5 |
| USP40 | RNF6 |
| TRAF6 | RNF7 |
| TRAF2 | RNF8 |
| USP45 | RNFT1 |
| RIPK1 | RNFT2 |
| ENY2 | RSPRY1 |
| OTUD6A | SCAF11 |
| USP17L15 | SH3RF1 |
| MDM2 | SH3RF2 |
| OTULIN | SH3RF3 |
| USP17L4 | SHPRH |
| USP17L8 | SIAH1 |
| STAMBP | SIAH2 |
| ATXN3L | SIAH3 |
| IKBKG | SMURF1 |
| OTUD4 | SMURF2 |
| RPS27A | STUB1 |
| ADRM1 | SYVN1 |
| MINDY1 | TMEM129 |

| | |
|---|---|
| SPATA2 | TOPORS |
| ZC3H12A | TRAF2 |
| MAP3K7 | TRAF3 |
| NLRP3 | TRAF4 |
| TRAF3 | TRAF5 |
| MYC | TRAF6 |
| MYSM1 | TRAF7 |
| MINDY2 | TRAIP |
| USP39 | TRIM10 |
| HIF1A | TRIM11 |
| | TRIM13 |
| | TRIM15 |
| | TRIM17 |
| | TRIM2 |
| | TRIM21 |
| | TRIM22 |
| | TRIM23 |
| | TRIM24 |
| | TRIM25 |
| | TRIM26 |
| | TRIM27 |
| | TRIM28 |
| | TRIM3 |
| | TRIM31 |
| | TRIM32 |
| | TRIM33 |
| | TRIM34 |
| | TRIM35 |
| | TRIM36 |
| | TRIM37 |
| | TRIM38 |
| | TRIM39 |
| | TRIM4 |
| | TRIM40 |
| | TRIM41 |
| | TRIM42 |
| | TRIM43 |
| | TRIM43B |
| | TRIM45 |
| | TRIM46 |
| | TRIM47 |
| | TRIM48 |
| | TRIM49 |
| | TRIM49B |

TRIM49C
TRIM49D1
TRIM5
TRIM50
TRIM51
TRIM52
TRIM54
TRIM55
TRIM56
TRIM58
TRIM59
TRIM6
TRIM60
TRIM61
TRIM62
TRIM63
TRIM64
TRIM64B
TRIM64C
TRIM65
TRIM67
TRIM68
TRIM69
TRIM7
TRIM71
TRIM72
TRIM73
TRIM74
TRIM75P
TRIM77
TRIM8
TRIM9
TRIML1
TRIML2
TRIP12
TTC3
UBE3A
UBE3B
UBE3C
UBE3D
UBE4A
UBE4B
UBOX5
UBR1

UBR2
UBR3
UBR4
UBR5
UBR7
UHRF1
UHRF2
UNK
UNKL
VPS11
VPS18
VPS41
VPS8
WDR59
WDSUB1
WWP1
WWP2
XIAP
ZBTB12
ZFP91
ZFPL1
ZNF280A
ZNF341
ZNF511
ZNF521
ZNF598
ZNF645
ZNRF1
ZNRF2
ZNRF3
ZNRF4
ZSWIN2
ZXDC

Supplementary Table S2. The antibodies and agents used in the study.

| Antibody/Agent | Assay | Catalog | Origin | Dilution/Concentration | Incubation period |
|---|---|---|---|---|---|
| B3GNT6 | WB | 21291-1-AP | Proteintech | 1/1000 | overnight, 4 °C |
| | IF | | | 1/500 | overnight, 4 °C |
| MUC1 | WB | TB5364 | Abmart | 1:1000 | overnight, 4 °C |
| E-Cadherin | WB | 2087 | Protein | 1:20000 | overnight, 4 °C |

| | | | | | |
|---|---|---|---|---|---|
| | | 4-1-AP | tech | | |
| N-Cadherin | WB | TA4039 | Abmart | 1:1000 | overnight, 4 °C |
| ZO-1 | WB | 21773-1-AP | Protein tech | 1:10000 | overnight, 4 °C |
| Vinculin | WB | 26520-1-AP | Protein tech | 1/40000 | overnight, 4 °C |

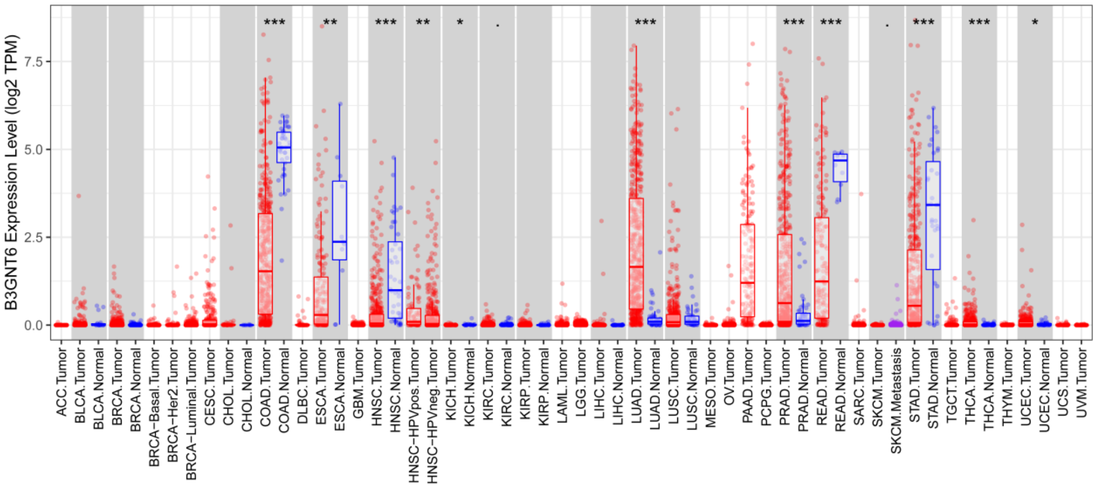


Supplementary Figure S1 The expression of B3GNT6 in pan-cancer.

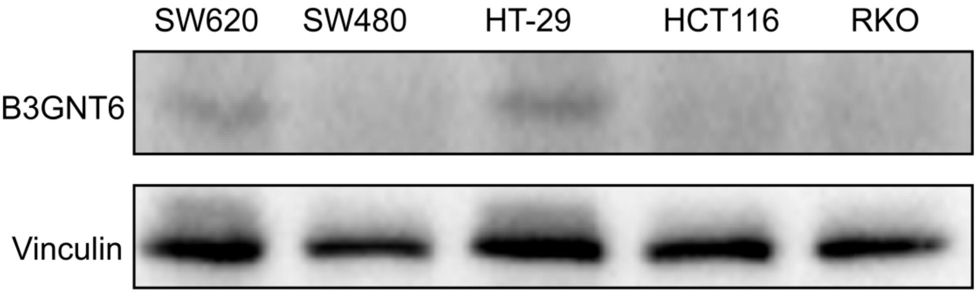


Supplementary Figure S2 B3GNT6 expression in a variety of colorectal cancer cell lines.

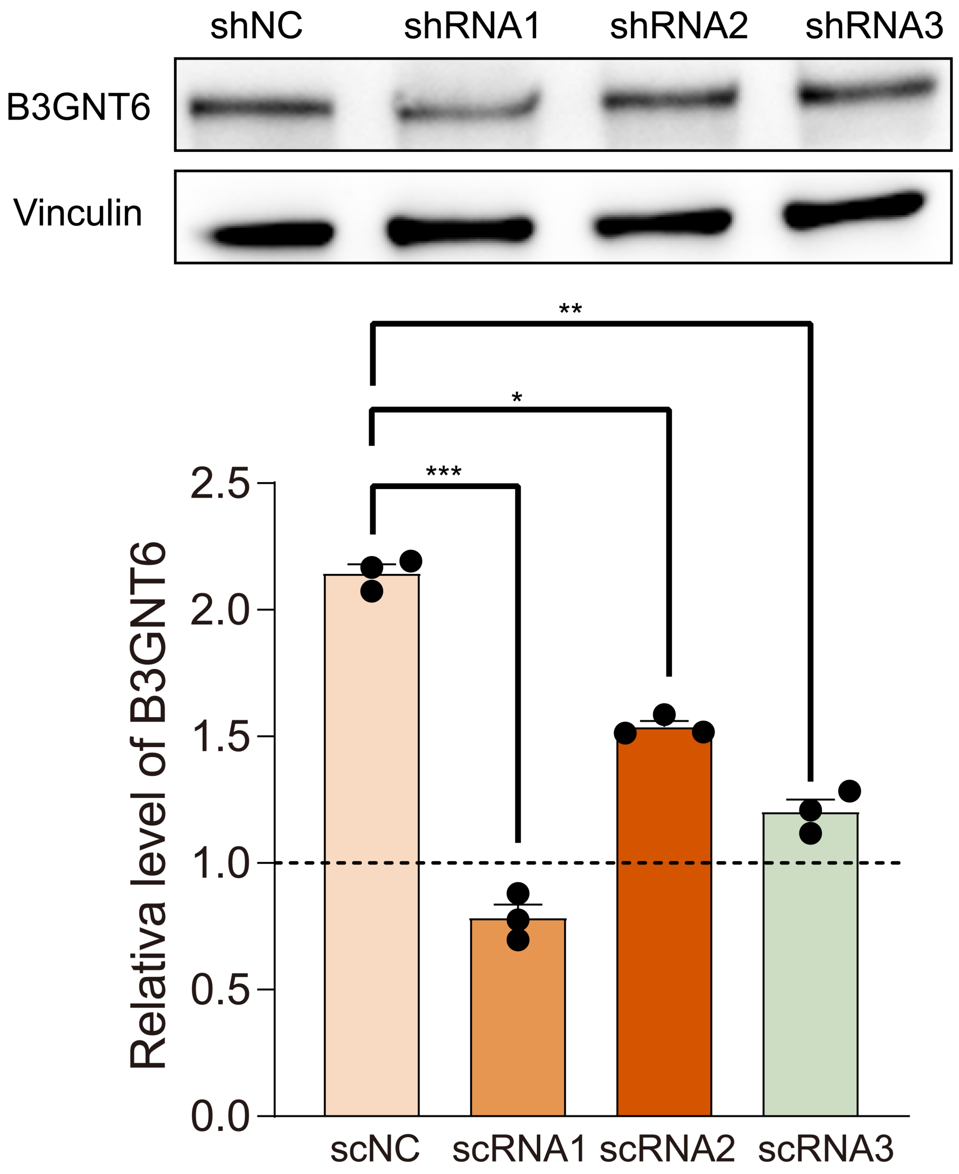


Supplementary Figure S3 B3GNT6 knockdown screening.
